# Protection of c-Fos from autophagic degradation by PRMT1-mediated methylation fosters gastric tumorigenesis

**DOI:** 10.7150/ijbs.85126

**Published:** 2023-07-15

**Authors:** Eunji Kim, Laily Rahmawati, Nur Aziz, Han Gyung Kim, Ji Hye Kim, Kyung-Hee Kim, Byong Chul Yoo, Narayana Parameswaran, Jong-Sun Kang, Hoon Hur, Balachandran Manavalan, Jongsung Lee, Jae Youl Cho

**Affiliations:** 1 Department of Integrative Biotechnology and Biomedical Institute for Convergence at SKKU (BICS), Sungkyunkwan University, Suwon 16419, Republic of Korea.; 2 R&D Center, Yungjin Pharmaceutical Co, Suwon 16229, Republic of Korea.; 3 Emergency Department, Hermina Hospital Tangkubanprahu, Malang 65119, Indonesia.; 4 Pharmacy Program, Faculty of Science and Engineering, Universitas Ma Chung, Malang 65151, Indonesia.; 5 Proteomic Analysis Team, Research Institute, National Cancer Center, Goyang 10408, Republic of Korea.; 6 Division of Translational Science, Research Institute, National Cancer Center, Goyang 10408, Republic of Korea.; 7 Department of Physiology and Division of Pathology, Michigan State University, East Lansing, Michigan 48824, USA.; 8 Department of Molecular Cell Biology, Single Cell Network Research Center, Sungkyunkwan University School of Medicine, Suwon 16419, Republic of Korea.; 9 Department of Surgery, Department of Biomedical Sciences, Ajou University School of Medicine, Suwon 16499, Republic of Korea.

**Keywords:** autophagy, c-Fos, gastric cancer, PRMT1, protein methylation, tumorigenesis

## Abstract

Both AP-1 and PRMT1 are vital molecules in variety of cellular progresssion, but the interaction between these proteins in the context of cellular functions is less clear. Gastric cancer (GC) is one of the pernicious diseases worldwide. An in-depth understanding of the molecular mode of action underlying gastric tumorigenesis is still elusive. In this study, we found that PRMT1 directly interacts with c-Fos and enhances AP-1 activation. PRMT1-mediated arginine methylation (mono- and dimethylation) of c-Fos synergistically enhances c-Fos-mediated AP-1 liveliness and consequently increases c-Fos protein stabilization. Consistent with this finding, PRMT1 knockdown decreases the protein level of c-Fos. We discovered that the c-Fos protein undergoes autophagic degradation and found that PRMT1-mediated methylation at R287 protects c-Fos from autophagosomal degradation and is linked to clinicopathologic variables as well as prognosis in stomach tumor. Together, our data demonstrate that PRMT1-mediated c-Fos protein stabilization promotes gastric tumorigenesis. We contend that targeting this modification could constitute a new therapeutic strategy in gastric cancer.

## Introduction

Protein arginine methyltransferases (PRMTs) modulate carrying a methyl group (CH_3_) to an arginine residue in proteinaceous substrates as a posttranslational modification 1,2. In addition to being identified in histone substrates, PRMTs have been discovered in many nonhistone substrates, including those in the nucleus, cell membrane, and cytoplasm 3-5. Members of the PRMTs are classified into 4 types derived from the type of arginine methylation catalyzed 6. PRMT1 is a type I PRMT, which catalyzes the formation of monomethylated arginine (omega-*N^G^*-monomethylarginine [MME-R]) and asymmetric *N^G^*,*N^G^*-dimethylarginine (ADME-R). Among the types of PRMTs, PRMT1 is the predominant type in mammals and accounts for >80% of PRMT activity 3. Hence, the various roles of PRMT1 in various processes and pathological conditions, such as inflammation, oxidative stress, cancer, and lymphocyte function, have been investigated 7-12.

The activator protein 1 (AP-1) contains homo- or hetero-dimers composed of different subunits from the Jun, Fos, and ATF families 13-16. AP-1 activity is stimulated by a variety of ligands, including cytokines, chemokines, and oncogenic stimuli, to modulate numerous cellular processes. In addition, posttranslational modifications such as methylation and SUMOylation have been proved to alter the transcriptional capacity of AP-1 by affecting its subunits 17-21. Fos proteins are basic region-leucine zipper (bZIP) proteins that interact with other bZIP or Jun proteins to build an AP-1 complex. Numerous tissues or types of cells constitutively express c-Fos, which regulates downstream genes by heterodimerizing with Jun proteins to mediate proliferation, angiogenesis, invasion, and metastasis 22-27. Fos has been shown to be essential for autophagy induction and is expressed at truly lower levels in melanoma tissues than in the counterpart nontumor tissues 23. In addition, c-Fos-mediated transcriptional activation is regulated by PRMT4/CARM1, which is involved in cancer and other diseases 28. However, i whether the c-Fos protein is affected or methylated by other PRMTs and how c-Fos arginine methylation influences disease development are unknown.

Gastric cancer (GC) has the highest mortality rate among malignant tumors and is one of the central causations of cancer-pertinent death globally, with a five-year alive rate of 20% 29-31. Despite advances in surgery and other therapies, patients with GC continue to have a poor prognosis and low chemosensitivity after surgical resection 29,32. Suppression of metastasis is key to increasing the survival rate of stomach cancer patients; thereupon, it is crucial to fully comprehend the fundamental pathophysiological and molecular events of GC invasion and metastasis. Both c-Fos and PRMT1 are important in cellular processes, and an interesting question is whether their interaction participates in cellular functions. Research on the function of particular PRMT family members in GC development is scarce. Such information is essential for the reasonable development of PRMT modulators for the remedy of cancerous diseases.

In this study, we focused on the c-Fos AP-1 subunit and investigated the function of PRMT1 in regulating c-Fos activity. We also identified the methylated arginine sites of c-Fos and reported how this methylation affected underlying mechanisms in GC cells. Notably, PRMT1 methylated c-Fos to modulate its protein stability via autophagy blockade, which supported gastric tumorigenesis. Together, our studies support an important role for PRMT1 in c-Fos and AP-1 regulation; specifically, inhibiting this axis might be an approach to treat GC.

## Materials and Methods

### Chemicals and antibodies

MG132, 3-methyladenine (3-MA), bovine serum albumin (BSA), cycloheximide (CHX), polyethylenimine (PEI), and 4',6-diamidino-2-phenylindole (DAPI) (chemical identifiers: 1211877-36-9, 5142-23, C7698, 9002-98-6, and 11024-24-1, respectively) were procured from Sigma. Luciferase-encoded DNA constructs containing AP-1 response elements were attained from Promega (Shanghai, China). The Myc-c-Fos, Flag-c-Fos, Flag-PRMT1, and EGFP-PRMT5 plasmids were constructed in our laboratory; the EGFP-PRMT1 and EGFP-PRMT3 plasmids were kind gifts from Prof. Kim (Sookmyung Women's University); and the pLKO.1 vector was obtained from Addgene (10878; deposited by David Root). Proteinase K (KB-0111) was acquired from Bioneer (Daejeon, Republic of Korea). The following primary antibodies were used: anti-LC3B, anti-MME-R, anti-ADME-R, anti-Myc, and anti-Flag (3868, 8015, 13522, 2276, and 8146; Cell Signaling Technology, Beijing, China). Antibodies recognizing β-actin, c-Fos, and GFP (sc-166940, sc-47778, and sc-9996; Santa Cruz Biotechnology, Heidelberg, Germany) were obtained, and Alexa Fluor 405-labelled and Alexa Fluor 568-labelled secondary antibodies to mouse and rabbit immunoglobulin (Invitrogen, Carlsbad, CA, USA) (A-31553 and A-11011) were used for staining.

### Cell culture

HEK293T (CRL-1573; ATCC, Manassas, VA, USA), MKN45, and MKN1 cells (80103 and 80101; KCLB, Seoul, Republic of Korea) were grown in RPMI 1640 medium or DMEM (SH30243.01 or SH30027.01; HyClone Laboratories, Sungnam, Republic of Korea) supplemented with antibiotics and fetal bovine serum (FBS) (16000-044; Gibco Laboratories, Tokyo, Japan). These cells were nourished in a five percent CO_2_ moisturized incubator (37 °C) following a previously reported protocol 33.

### Constructs and mutagenesis

The expression constructs for c-Fos and PRMT5 were generated through PCR amplification, utilizing HEK293T cell cDNA as the template. A PRMT1 dominant negative (DN) mutant with mutation of 63VLD65 to 63AAA65 was also constructed with a Stratagene QuikChange mutagenesis kit (Stratagene, Seoul, Republic of Korea). Similarly, c-Fos methylated point mutants (R108K, R201K, R279K, R287K, and R287F) were generated using the same methodology. The nucleic acid primers employed for generating the site- or domain-targeted mutants are provided in **Table 1.**

### Lentivirus-mediated knockdown with short hairpin RNA (shRNA)

The plasmids containing shRNA sequences targeting PRMT1 were constructed following the protocols provided by Addgene (www.addgene.org). The pLKO.1 vector was used for constructing the plasmids containing the nontargeting scrambled shRNA sequence (TCCTAAGGTTAAGTCGCCCTCG) and the PRMT1 shRNA sequence (CCGGCAGTACAAAGACTACAA). Lentivirus production was carried out by transient transfection of HEK293T cells. The resulting lentiviruses were then utilized to infect cells, and subsequently, puromycin treatment was employed to select cells that were stably transduced with either shScramble or shPRMT1. The effectiveness of PRMT1 knockdown was verified by immunoblotting.

### DNA transfection and luciferase reporter assay

It is generally accepted that cells with a high transfection efficiency are required for luciferase assays. Since HEK293T cells are known to have a higher transfection efficiency than other cell lines, these cells were chosen for this work, as reported previously 34,35. To perform this assay, HEK293T cells were transfected in a 24-well plate with either empty vector or the specified plasmids (c-Fos, PRMT1, PRMT3, or PRMT5) at 0.25 μg per well. Additionally, Luc constructs were transfected at 0.25 μg per well along with β-galactosidase at 0.1 μg per well. Transfection was performed using polyethylenimine (PEI) following a previously reported method 36. The next day, the cells were retreated with FBS-containing medium or the desired compound and further incubated for an additional day. Following this treatment, the cells transfected with DNA were harvested, and the activity of the luciferase reporter was quantified by employing a Promega enzyme determination kit (E1500; Promega, Beijing, China).

### Preparation of whole-cell lysates and tissues; immunoblotting

We collected thirty pairs of stomach cancer tissues and normal adjacent tissues (NATs) from stomach cancer patients who received surgery at AUH (Ajou University Hospital, Suwon, Republic of Korea). The specimens were sourced from the AHBRB (Ajou Human Bio-Resource Bank). All patients agreed the use and storage of their samples. The project was orchestrated with strict adherence to the ethical guidelines and permission from the Institutional Review Board of Ajou University Hospital (AJIRB-BMRKSP-19-059), as previously mentioned 30,33. The tumor tissue and NAT specimens obtained from GC patients were crushed under liquid nitrogen, while cells were washed with PBS (B2814; Samchun Pure Chemical, Pyeongtaek, Republic of Korea). The washed cells were collected, centrifuged, and lysed in buffer, as described in previous reports 37,38. The upper layer obtained after centrifugation was collected and utilized for Western blot analysis. Antibodies specific for Myc, Flag, GFP, total c-Fos, LC3B, MME-R, ADME-R, and β-actin were used for immunoblotting 39.

### Immunoprecipitation

Cell lysates containing 1,000 μg protein were prepared and reacted with 5 μL of the primary antibodies overnight with stirring in a refrigerator. After the incubation period, immune complexes were incubated with protein A- or G-coupled Sepharose beads (40 μL, 50% v/v) with rotation (4 h, 4 °C). Next, the immune complexes were boiled and subjected to immunoblotting to measure the protein levels following the methodology previously reported 40-42.

### Immunofluorescence staining and image analysis

Adherent HEK293T cells expressing Myc-c-Fos and/or EGFP-PRMT1 were fixed with a solution of 3.7% paraformaldehyde, permeabilized by treating 1% Triton X-100, and incubated with 1% BSA. After staining with primary and secondary antibodies, the cells were immersed in Hoechst staining solution (diluted 1:1000). To counterstain nuclear DNA, DAPI was utilized 43. Confocal imaging was conducted with a laser scanning microscope (LSM 700, Zeiss, Pendleton, IN, USA) 44,45.

### mRNA analysis by semiquantitative RT‒PCR or quantitative RT-PCR

The mRNA expression status of c-Fos from Myc-c-Fos- and/or EGFP-PRMT1-transfected HEK293T cells was scrutinized by both semiquantitative reverse transcription polymerase chain reaction (RT‒PCR) 46 and quantitative real-time RT‒PCR (qRT‒PCR) in accordance with previously reported methods 47,48.

### Protease protection assay

The protein level in proteasomes was measured by a protease protection assay in accordance with a previous report 49. Myc-c-Fos- and/or EGFP-PRMT1-transfected HEK293T cells were incubated with digitonin solution (6.5 μg/mL, BN2006; ThermoFisher, Heysham, UK), mixed with protease (proteinase K) and Triton X-100, and were then scrutinized by electrophoresis.

### LC‒MS/MS

Myc-c-Fos-overexpressing cells were harvested and prepared for immunoprecipitation using an anti-Myc antibody. Proteins in the immunoprecipitated samples were then separated by SDS‒PAGE. Subsequently, excised gel samples were digested with trypsin (37 °C, overnight). After digestion, the samples were lyophilized, reconstituted, and fractionated. The fractions were then analyzed using strong cation exchange liquid chromatography (SCXLC) in combination with mass spectrometry.

### QuantSeq 3' mRNA sequencing

PRMT1-knockdown MKN45 cells and MKN45 cells with reconstitution of PRMT1 were harvested, and total RNA was isolated with TRIzol reagent. RNA sequencing and data interpretation were managed by E-Biogen (Daejeon, Republic of Korea) on a NextSeq 500 instrument from Illumina, Inc. (Seoul, Republic of Korea). The library was formulated with a QuantSeq 3' mRNA-Seq Library Prep Kit (Lexogen, Inc., Seoul, Korea). The sequencing data were analyzed with Excel-Based Differentially Expressed Gene Analysis (ExDEGA) GraphicPlus v2.0 by filtering DEGs based on a threshold fold change of greater than 2. In addition, gene set enrichment analysis (GSEA) of chosen genes was delved with GSEA software 33.

### Analysis of microarray and RNA-seq data from a publicly available database

The GSE66229 50, GSE26899 50, GSE54129 (unpublished), and GSE79973 51 datasets established with gene expression patterns of tumor and normal tissues were acquired from Gene Expression Omnibus (GEO). Unified STAD normal, STAD tumor, and healthy GTEx stomach tissue data were downloaded from Schultz et al. 52. Nonpairwise comparisons of these data sets were evaluated with R version 4.2.1 (R Foundation for Statistical Computing, Indianapolis, IN, USA).

### Multiple sequence alignment

The FASTA protein sequences of PRMT1 from various types of Genus and species were acquired from the UniProt database. Alignment of DNA sequences was carried out in Jalview 2.11.1.4 using ClustalW with default parameters.

### Cell proliferation assay

DNA construct (c-Fos WT or its mutants)-transfected, PRMT1-overexpressing and PRMT1-knockdown MKN45 cells were incubated for indicated days. Cell proliferation was quantified by an MTT assay in accordance with previously reported protocols 30.

### Wound healing assay

MKN45 cells transfected with empty vector or plasmids containing PRMT1 DNA together with the c-Fos WT or c-Fos R287K plasmid were seeded (5.5 × 10^5^ cells/well) in a culture (12-well) plate. The cells were cultured overnight, scratched with a tip, and imaged after 0, 24, 48, and 72 h. The wound closure rate was determined with ImageJ of National Institute of Health (NIH, USA).

### Colony formation assay

MKN45 cells were transfected with empty vector or the indicated plasmids (PRMT1 and c-Fos WT or c-Fos R287K) for 24 h using Lipofectamine 2000 (11668-019; Invitrogen) and thereafter seeded (0.5 × 10^3^ cells per well) in a culture plate with six wells. After 10 days of culture, the cells were immersed in fixation solution (4% paraformaldehyde, 15 min). Subsequently, the cell aggregates were tinted with staining solution (0.5% crystal violet, C3886; Sigma). The stained cells in three random fields per well were counted and analyzed with a microscope connected to a camera and ImageJ.

### Invasion assay

PRMT1-overexpressing MKN45 cells or c-Fos-transfected and PRMT1-knockdown MKN45 cells (5 × 10^4^ cells/well) were plated in the top layer of a Transwell chamber with a membrane-permeable polycarbonate filter coated with BD Biosciences Matrigel (356237; San Diego, CA, USA) in Opti-MEM (11058021; Gibco Laboratories). The bottom compartment of the 24-well plate was filled with RPMI 1640 medium as a chemoattractant. Following 24 h of incubation, invaded cells from the upper compartment were fixed by 4% paraformaldehyde treatment and tinted with hematoxylin (ab220365; Abcam, Cambridge, US) and eosin Y solution (HT110116; Sigma‒Aldrich). The invaded cells in three random areas per well were counted and analyzed with a microscope connected to a camera and ImageJ.

### Statistical analysis

Statistical values (*P* < 0.05) were figured with Student's *t*, the Mann-Whitney *U*, or R (version 4.2.1) tests, and data visualization was achieved with SigmaPlot 11.0. The Kaplan‒Meier method was specifically applied for cancer patient survival rate profile analysis.

## Results

### c-Fos directly interacts with PRMT1

**Figure 1:**
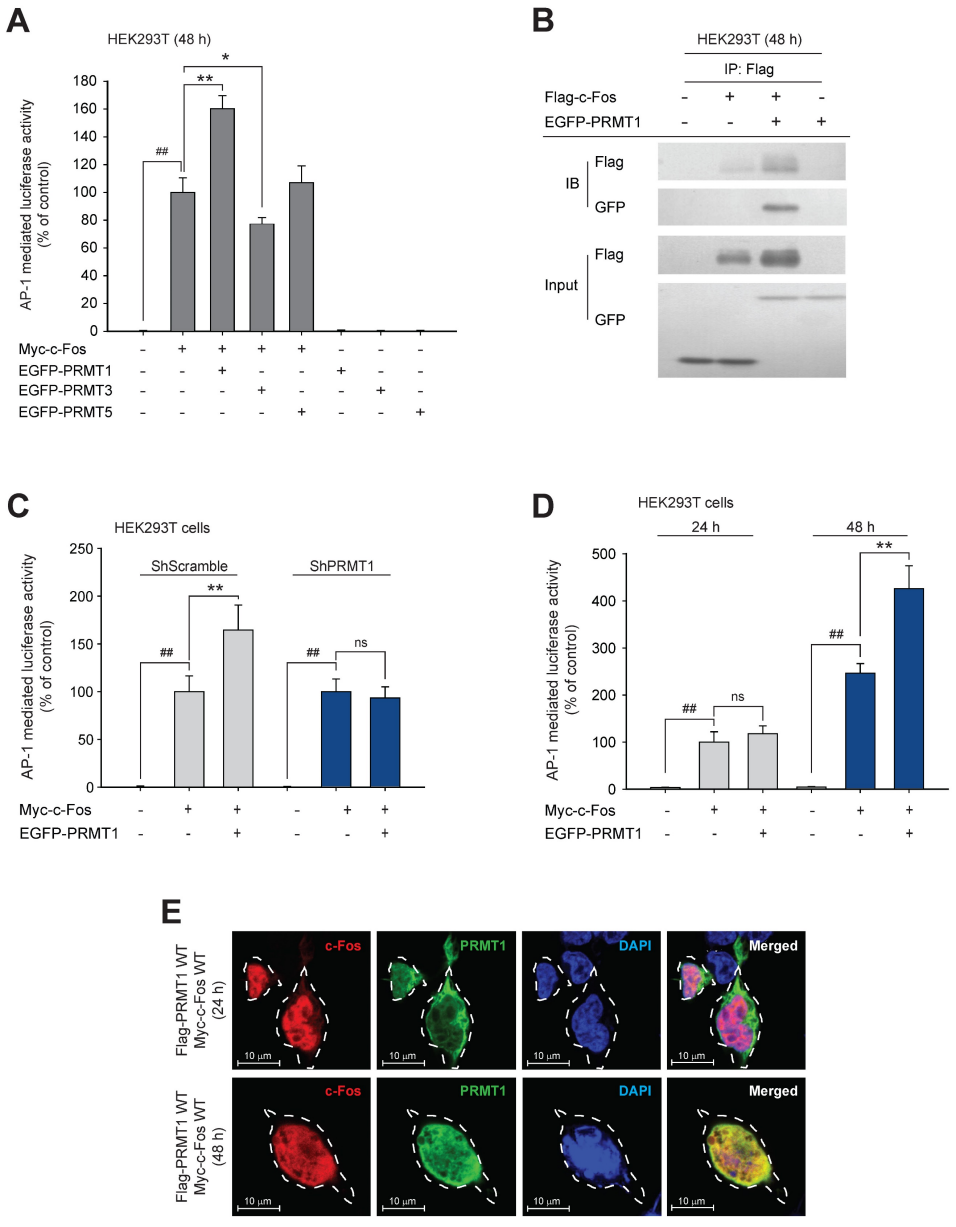
PRMT1 regulates c-Fos activity. (A) c-Fos and PRMT1, 3, or 5 were cotransfected into HEK293T cells for 48 h. c-Fos-induced AP-1-mediated luciferase reporter liveliness was quantified by a luminescence detector. (B) HEK293T cells overexpressing Flag-c-Fos and EGFP-PRMT1 were lysed for an immunoprecipitation assay. Flag and GFP levels were measured by immunoblotting of the anti-Flag immunoprecipitate obtained from whole-cell lysates of HEK293T cells. c-Fos-induced AP-1-mediated luciferase activity was measured in (C) shScramble- and shPRMT1-expressing cells and in (D) HEK293T cells transfected with Myc-c-Fos and EGFP-PRMT1 for the indicated times. Cells were lysed with buffer, and luminescence was measured. (E) HEK293T cells were transfected with Flag-PRMT1 and Myc-c-Fos for the indicated times. At the endpoint, cells were permeabilized and fixed for confocal analysis. Alexa Fluor 568 and Hoechst were used for recognition of c-Fos (indicated in red, c-Fos) and nuclei (indicated in blue, DAPI), respectively. ^##^*P* < 0.01 versus the normal and *P < 0.05 and **P < 0.01 versus the c-Fos alone.

To identify whether c-Fos activity can be regulated by methyltransferases, we first performed mass spectrometry using c-Fos-immunoprecipitated protein samples (**Fig. S1A**). The results showed that RNA methyltransferases, DNA methyltransferases, and protein methyltransferases interacted with the c-Fos protein (**Table 2**). Eleven protein methyltransferases, including the protein arginine methyltransferase (PRMT) family and histone-lysine methyltransferases, were detected. Among the detected methyltransferases, we focused on PRMTs because they are located in both the nucleus and cytosol, which is similar to the cellular distribution of c-Fos. To study the effects of PRMTs on c-Fos function, we used a luciferase assay to ascertain whether c-Fos-mediated AP-1 activity is influenced by PRMTs. We observed that only PRMT1 dramatically and synergistically enhanced c-Fos-induced AP-1 activity, whereas transfection of PRMTs alone did not affect AP-1 activation (**Fig. 1A**). Unlike activity mediated by the other AP-1 subunits, c-Jun-mediated activity was not enhanced by PRMT1 (**Fig. S1B**). The increase in c-Fos-induced AP-1 activity was further evaluated by an immunoprecipitation assay, and we found that PRMT1 formed a complex with c-Fos (**Fig. 1B**), suggesting that PRMT1 interacts with the c-Fos transcription factor for specifically adjusting c-Fos-induced AP-1 activity. To determine whether activation of AP-1 is enhanced specifically by PRMT1, other protein methyltransferases were analyzed. Protein histidine methyltransferase (PHMT) and lysine methyltransferases (FAM86A and CAMKMT) did not affect c-Fos-mediated AP-1 activity (**Fig. S1C**). Interestingly, c-Fos-induced AP-1 liveliness was DNA-dose dependently upregulated by transfection of PRMT1 (**Fig. S1C**). Furthermore, knockdown of PRMT1 did not affect AP-1 activity induced by c-Fos (**Fig. 1C**). In a time-course assay, PRMT1-mediated c-Fos/AP-1 activation was observed after 48 h (but not after 24 h) of transfection (**Fig. 1D**). Concordant with this finding, in the immunohistochemistry and confocal microscopy experiments, colocalization of PRMT1 and c-Fos was verified in the nucleus 48 h post-transfection (**Fig. 1E**). This suggests that PRMT1 likely has a time-dependent synergistic effect on c-Fos activity.

### PRMT1 methylates the c-Fos protein

**Figure 2:**
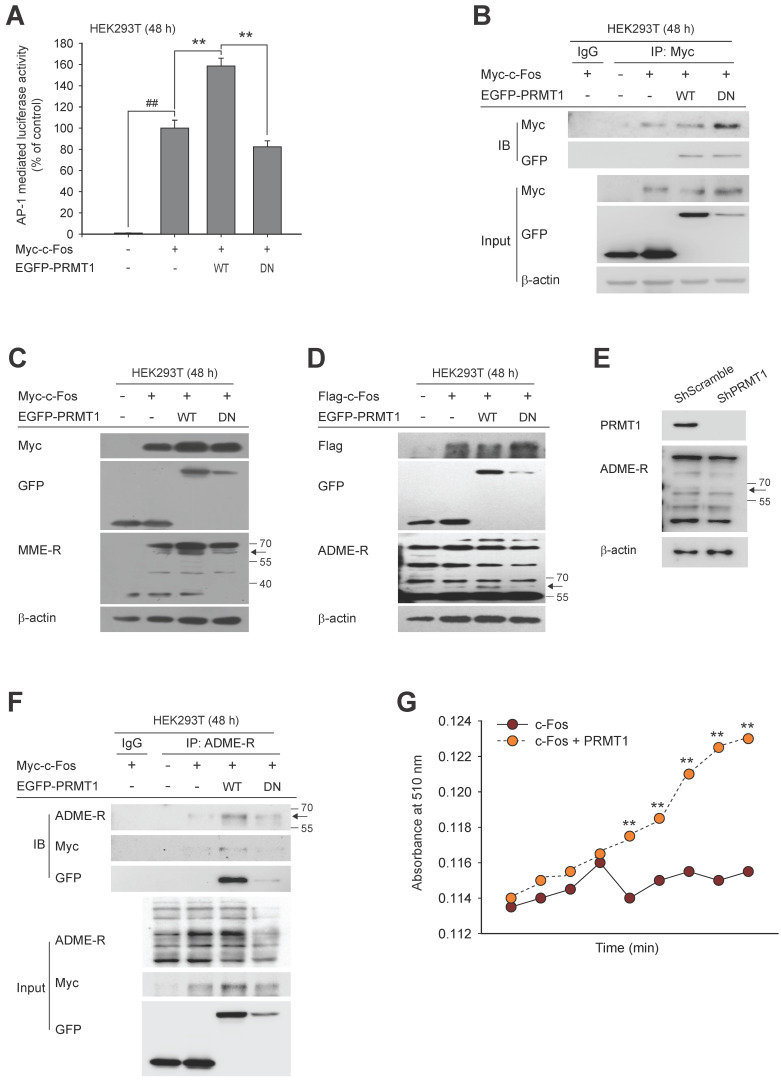
PRMT1 methylation-dependent regulation of c-Fos activity. (A) Myc-c-Fos and EGFP-PRMT1 WT or DN were cotransfected into HEK293T cells for 48 h; then, c-Fos-mediated AP-1 activity was measured by a luciferase assay. (B) Immunoprecipitation was performed with whole-cell lysates prepared from HEK293T cells transfected with Myc-c-Fos, EGFP-PRMT1 WT, or EGFP-PRMT1 DN. The levels of Myc and GFP were measured by immunoblotting. (C) Monomethylated arginine levels and (D) dimethylated arginine, Flag, and GFP levels were measured by immunoblotting in whole-cell lysates of HEK293T cells transfected with Myc-c-Fos and EGFP-PRMT1. (E) Asymmetric dimethylarginine (ADME-R) was detected in whole-cell lysates prepared from shScramble- or shPRMT1-expressing cells by immunoblotting. (F) ADME-R, Myc, and GFP levels were measured by immunoblotting in the ADME-R immunoprecipitate from whole-cell lysates of HEK293T cells transfected with Myc-c-Fos and EGFP-PRMT1 for 48 h. (G) Ectopic c-Fos or PRMT1 was immunoprecipitated, and these proteins were incubated with SAM following the manufacturer's instructions to measure the methyl-accepting capacity of c-Fos. ^##^*P* < 0.01 versus the normal and **P < 0.01 versus the c-Fos alone.

PRMT1 has been shown to catalyze the transfer of methyl groups to substrates, leading to diverse cellular processes 53,54. However, there is no evidence that c-Fos is a methylation substrate for PRMT1. We hypothesized that the synergistic effect of PRMT1 on c-Fos activity is contingent on the methylation of c-Fos by PRMT1. Thus, we next evaluated whether PRMT1 can methylate c-Fos. A dominant negative (DN) mutant of PRMT1 without methyltransferase activity 55,56 was constructed (**Fig. S2**) and used to evaluate both the effect of PRMT1 on c-Fos-mediated AP1 activity via a luciferase assay and the interaction between PRMT1 and c-Fos using immunoprecipitation. As predicted, overexpression of the DN mutant did not enhance c-Fos-mediated AP-1 activation (**Fig. 2A**). However, the interaction between c-Fos and PRMT1 was not altered by the DN mutation (**Fig. 2B**). PRMT1 is classified as a type I PRMT that can generate both MME-R and ADME-R 63,39.

To determine whether the interaction between PRMT1 and c-Fos leads to methylation of c-Fos, we sought to detect methylated arginine residues using antibodies specific for MME-R and ADME-R. When PRMT1 was cotransfected with c-Fos, both forms of methylated arginine residues were increased at the molecular weight corresponding to c-Fos (approximately 60 kDa) (seen in **Fig. 2C** and** D**). On the other hand, PRMT1 DN mutant-transfected cells showed very low levels of methylated c-Fos. In addition, a low level of methylated c-Fos was observed in PRMT1-knockdown cells (**Fig. 2E**). In the immunoprecipitation assay, we analyzed immunoprecipitated lysates using an anti-ADME-R antibody and detected a putative band corresponding to c-Fos at 55-70 kDa in PRMT1 wild-type (WT) and c-Fos-transfected cell lysates. The intensity of the ADME-R-modified c-Fos band was nearly zero in cells expressing the PRMT1 DN mutant that were cotransfected with c-Fos (**Fig. 2F**). These data demonstrate that c-Fos and PRMT1 were coimmunoprecipitated and that c-Fos was likely dimethylated asymmetrically by PRMT1. Next, we performed an *in vitro* methylation assay to confirm direct methylation of c-Fos by PRMT1. **Fig. 2G** shows that the presence of PRMT1 was able to elevate the methylation level of c-Fos. Collectively, such findings imply that c-Fos is undergoes arginine dimethylation catalyzed by PRMT1, which enhances AP-1 activity induced by c-Fos.

### PRMT1 regulates c-Fos protein stability

**Figure 3:**
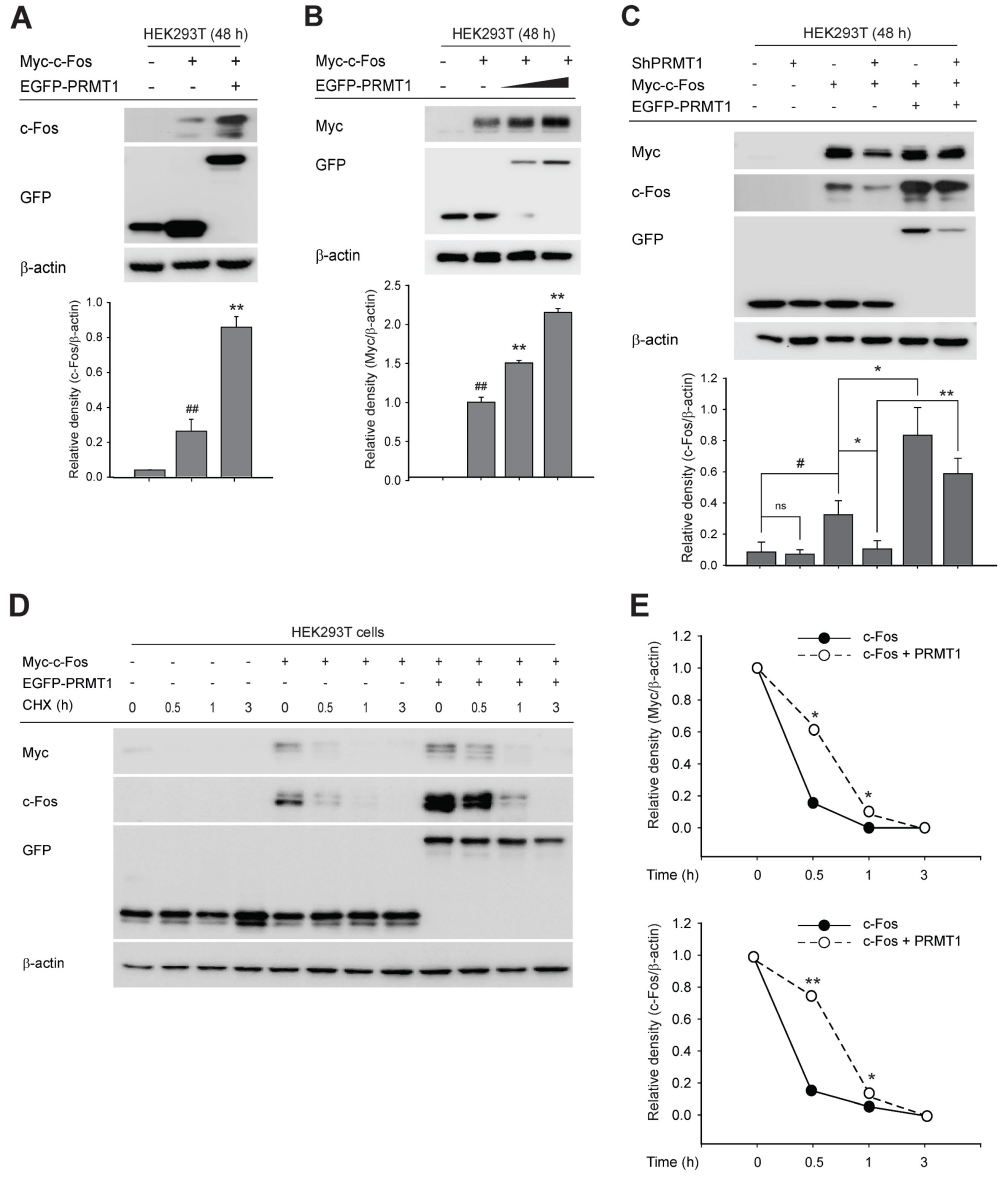
PRMT1 regulates c-Fos protein stability. (A) Myc-c-Fos and EGFP-PRMT1 were cotransfected into HEK293T cells for 48 h. Harvested cells were used for immunoblot analysis of c-Fos and GFP levels. (B) Myc-c-Fos and EGFP-PRMT1 were transfected at gradually increasing concentrations into HEK293T cells for 48 h. Harvested cells were used for immunoblot analysis of Myc and GFP levels. The relative density of the Myc protein band was determined by ImageJ. (C) Myc-c-Fos and EGFP-PRMT1 were transfected into HEK293T WT or PRMT1-knockdown cells. Harvested cells were used for immunoblot analysis of c-Fos, GFP, and Myc levels. A cycloheximide (CHX) chase assay was performed with Myc-c-Fos/EGFP-PRMT1-overexpressing HEK293T cells by treatment with 30 μg/mL CHX for the indicated times (0-3 h). (D) Whole-cell lysates were prepared for immunoblot analysis, and (E) the relative densities of the Myc and c-Fos protein bands were determined by ImageJ.

We next focused on the mechanism by which PRMT1 regulates c-Fos-mediated AP1 activity. Our initial experiments ruled out an effect of PRMT1 on c-Fos gene expression (**Fig. S3A** and **S3B**), phosphorylation of MAPK (**Fig. S3C** and **S3D**) or c-Fos (**Fig. S3E**), and dimerization of c-Fos and c-Jun (**Fig. S3F**). Although PRMT1 overexpression did not alter c-Fos gene expression (**Fig. S3A** and **S3B**), the protein level of the c-Fos, which shows a wide range of molecular weights due to its posttranslational modifications, such as ubiquitination, phosphorylation, and *S*-nitrosylation 57-59, was magnified by PRMT1 transfection (**Fig. 3A**) and was synergistically and concentration-dependently increased (**Fig. 3B**). Consistent with this finding, knockdown of PRMT1 blocked c-Fos expression, which was restored when the c-Fos expression plasmid was transfected (**Fig. 3C**). To examine whether PRMT1 regulates c-Fos protein stability, a cycloheximide (CHX) chase assay was carried out. CHX has been widely used for protein stability assays because it blocks protein translation 60-62. In this experiment, CHX was added 48 h after transfection of the c-Fos and PRMT1 plasmids. As expected, the c-Fos protein level decreased over a period of 3 h. Interestingly, the PRMT1/c-Fos cotransfected group had a higher c-Fos level than the c-Fos control-transfected group (**Fig. 3D** and** E**). Together, these data demonstrate that PRMT1 regulates c-Fos activity by enhancing its protein stability.

### c-Fos protein stability is modulated by autophagy

**Figure 4:**
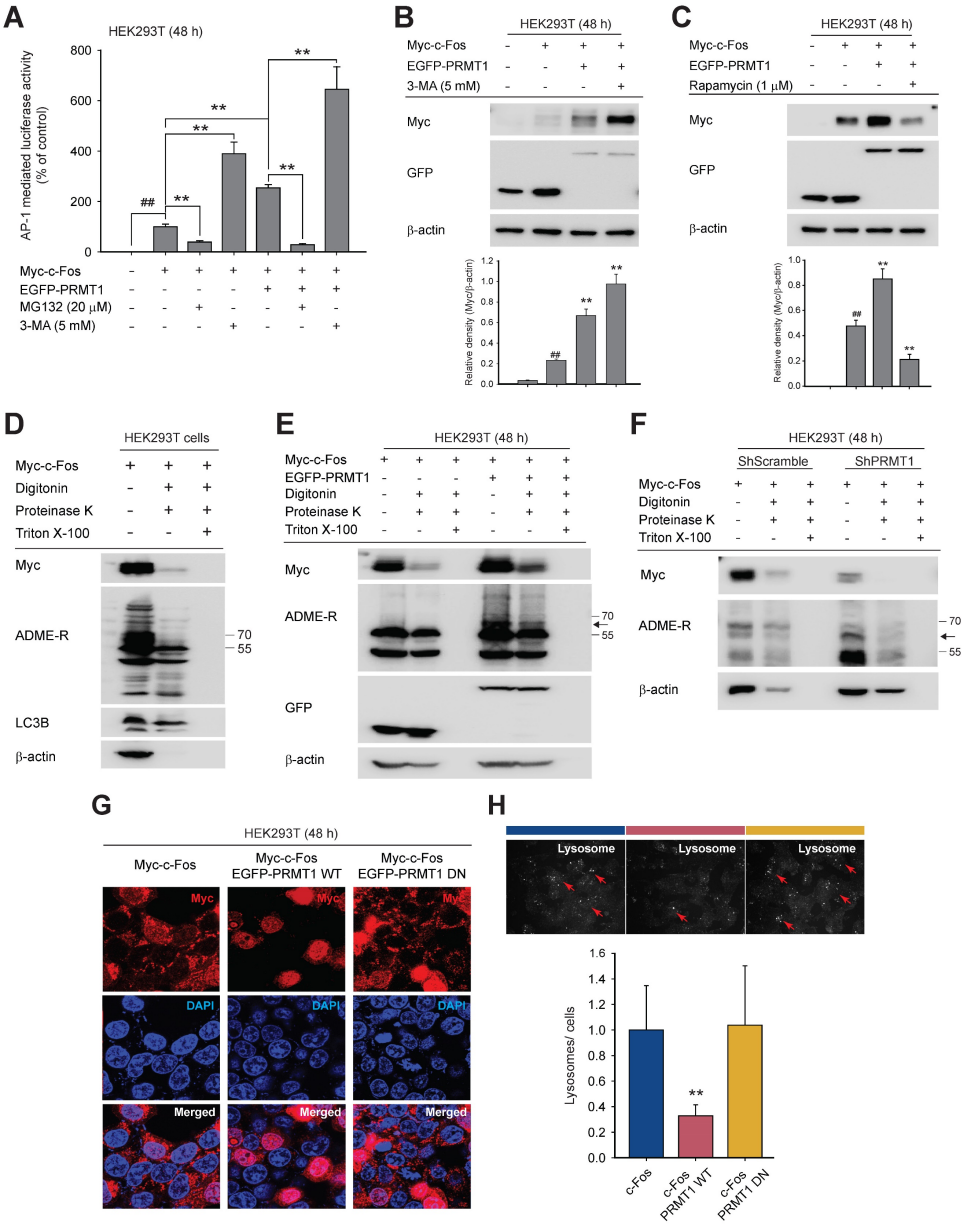
PRMT1 protects c-Fos from autophagic degradation. (A) HEK293T cells were transfected with Myc-c-Fos and EGFP-PRMT. A proteasome or autophagy inhibitor (MG132 or 3-MA, respectively) was added for an additional 24 h of incubation. AP-1-mediated luciferase reporter liveliness was quantified by a luminescence detector. (B and C) Myc-c-Fos- and EGFP-PRMT1-overexpressing HEK293T cells were treated with 3-MA (5 mM) or rapamycin (1 μM); then, the protein levels of Myc and GFP were measured by immunoblotting. (D) Autophagic degradation of c-Fos, ADME-R, and LC3B was detected in autophagosomes/lysosomes of HEK293T cells by a protease protection assay and immunoblot analysis. (E and F) Endosomes were isolated for a proteasome protection assay. With whole-cell lysates prepared from HEK293T cells (E) or PRMT1-knockdown cells (F), the protein levels of Myc, ADME-R, and GFP were measured by immunoblot analysis. (G) HEK293T cells were cotransfected with Myc-c-Fos and EGFP-PRMT1 WT or DN for 48 h and then immunostained with Alexa Fluor 568 (for c-Fos) and Hoechst (for nuclear DNA), and the formation of puncta was observed by confocal microscopy. (H) HEK293T cells transfected with c-Fos and PRMT1 were incubated with LysoTracker following the manufacturer's manual. Lysosomes were identified by fluorescence microscopy. ^##^*P* < 0.01 versus the normal and **P < 0.01 versus the c-Fos alone or c-Fos/PRMT1 cotransfection group. Arrows (H) indicate lysosomes.

To decipher how PRMT1 regulates c-Fos stability, we used the protein degradation inhibitors MG132 (a proteasomal degradation inhibitor) and 3-MA (an autophagy inhibitor). Proteasomal degradation and autophagy are the two main mechanisms of protein degradation 63,64. The addition of 3-MA enhanced PRMT1-mediated c-Fos/AP-1 activity, whereas MG132 inhibited AP1 activity (**Fig. 4A**). We subsequently confirmed that 3-MA treatment increased the c-Fos protein level (**Fig. 4B**). Conversely, treatment with rapamycin, an autophagy inducer, decreased the c-Fos protein level (**Fig. 4C**) and reduced c-Fos-induced AP-1 activity (**Fig. S4A**).

During autophagic degradation, autophagosomes and lysosomes are formed and envelop degradable proteins. In this study, we isolated autophagosomes by a protease protection assay 49. The lysosomal location of c-Fos was identified (**Fig. 4D**), and we determined whether the concentration of c-Fos was affected by the expression level of PRMT1. Upon coexpression of PRMT1 and c-Fos, c-Fos accumulated in the proteasome, and methylated arginine was detected by immunoblotting (**Fig. 4E**). In contrast, when PRMT1 was knocked down, the c-Fos protein was rapidly degraded, and the intensity of the band corresponding to ADME-R-modified c-Fos was reduced (**Fig. 4F**). In addition, the LC3-II level was increased under PRMT1-knockdown conditions (**Fig. S4B**). Moreover, overexpression of c-Fos increased the number of puncta visualized by confocal microscopy (**Fig. 4G**). Moreover, the number of vacuoles was reduced in the presence of PRMT1 but was restored when PRMT1 DN was cotransfected. A similar pattern was observed in the LysoTracker assay (**Fig. 4H**). Collectively, these data demonstrate that c-Fos could be degraded via the autophagic manner and that PRMT1 could prevent c-Fos's autophagic degradation.

### Methylation of c-Fos on R287 protects it from autophagic degradation

**Figure 5:**
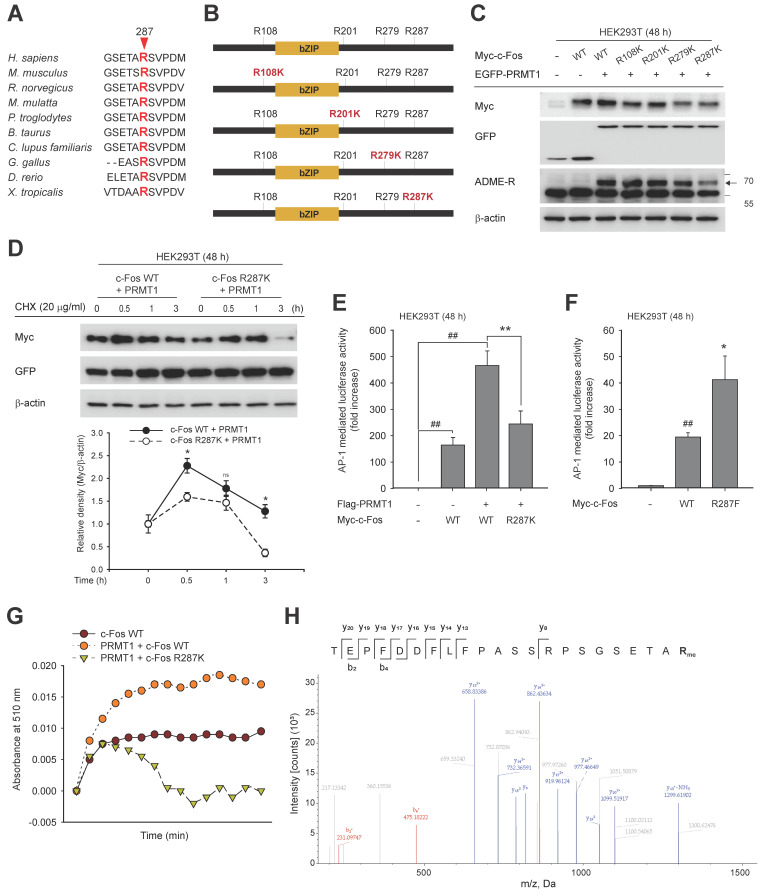
PRMT1 methylates the R287 residue of c-Fos. (A) Amino acid sequence alignment of c-Fos arginine methylation sites across species. (B) Diagram of c-Fos and its mutants (WT, R108K, R201K, R279K, and R287K). (C) c-Fos WT and its mutants were cotransfected with PRMT1 into HEK293T cells. Myc, GFP, and ADME-R levels were measured by immunoblot analysis. (D) A CHX chase assay was conducted in HEK293T cells cotransfected with PRMT1 and c-Fos WT or c-Fos R287K by CHX treatment (30 μg/mL) at the indicated times. Myc and GFP levels were measured by immunoblot analysis. (E and F) Myc-c-Fos WT, R287K, or R287F was transfected into HEK293T cells in the presence or absence of Flag-PRMT1 with the AP-1-Luc construct. AP-1 activation was then measured by a luminometer. (G) Lysates of cells cotransfected with PRMT1 and c-Fos WT or c-Fos R287K were subjected to immunoprecipitation, and the immunoprecipitated proteins were incubated with SAM for measurement of the methyl-accepting capacity by spectrometry. (H) Methylation of the R287 residue of c-Fos was detected by LC‒MS/MS. ^##^*P* < 0.01 versus the normal, *P < 0.05 and **P < 0.01 versus the c-Fos alone or c-Fos/PRMT1 cotransfection group.

By mass spectrometry, we identified the methylated arginine sites of c-Fos as R108, R201, R279, and R287. Many methylated sites of proteins are conserved among species 65,66. We compared these four sites among 10 species, including *Rattus norvegicus*, *Mus musculus*, and* Homo sapiens*, and found that all were highly conserved (**Fig. S5** and** Fig. 5A**). To identify the site methylated directly by PRMT1, arginine (R)-to-lysine (K) substitution mutants were generated as shown in **Fig. 5B**, and asymmetric dimethylarginine levels were evaluated. The translational states of c-Fos R279K and R287K were slightly decreased, but a reduced methylated arginine level was observed only in the R287K mutant (**Fig. 5C**). Protein stability was examined with c-Fos WT and R287K under co-expression of PRMT1. As exhibited in **Fig. 5D**, PRMT1 protected c-Fos WT against degradation, but the c-Fos R287K mutant protein was rapidly lost. Consistent with the results of the protein stability assay, reduced c-Fos R287K-induced activation of AP-1 and weak synergistic AP-1-medited induction of PRMT1 were also observed (**Fig. 5E**). To further strengthen these findings, a c-Fos methylation mimetic mutant, R287F, was constructed. Compared to c-Fos WT, the R287F mutant significantly enhanced AP-1 activity (**Fig. 5F**). Finally, we measured the c-Fos R287K methylation level. In line with the previous result (**Fig. 2G**), PRMT1 overexpression increased the c-Fos WT methylation level. However, the c-Fos R287K methylation level was decreased even in the presence of PRMT1 overexpression, suggesting that c-Fos R287K was not methylated (**Fig. 5G**). In addition, using mass spectrometry, we further verified methylation of c-Fos at R287 (**Fig. 5H**) and, as revealed in **Fig. 5A**, showed that c-Fos R287 is conserved across species, indicating that this regulatory mechanism is also conserved across species. Together, these results show that PRMT1 methylates c-Fos at R287 and protects c-Fos from autophagic proteolysis.

### PRMT1 is upregulated in GC and associated with clinicopathologic variables of GC

**Figure 6:**
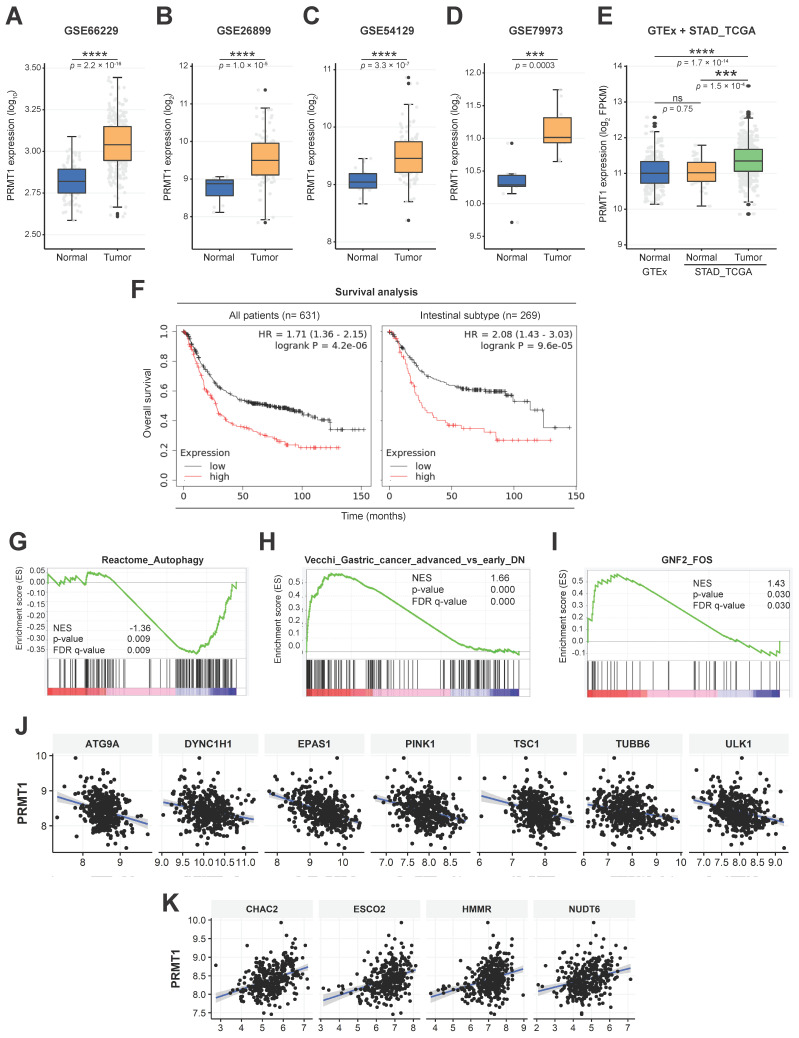
Gene expression profile of PRMT1 and survival rate of patients based on PRMT1 expression in GC. (A-D) PRMT1 gene expression in gastric normal vs. tumor tissues in several GEO datasets containing data from gastric cancer patients. (E) Comparison between PRMT1 gene expression in gastric normal tissues (STAD_TCGA+GTEx) vs. gastric tumor tissues (STAD_TCGA). (F) Overall survival analysis of gastric cancer patients based on the transcriptional level of PRMT1 was completed by employing Kaplan‒Meier Plotter (www.kmplot.com). (G-I) GSEA of autophagy genes, gene markers of advanced GC, and FOS target genes based on our RNA-seq data from PRMT1-reconstituted cells compared to shPRMT1 cells. (J and K) Visualization of the correlations of PRMT1 expression with that of several genes in the STAD tumor dataset.

To study the biological roles of PRMT1 and c-Fos, we analyzed PRMT1 expression data for a variety of cancers obtained from The Cancer Genome Atlas (TCGA) by employing TIMER. These analysis results showed higher expression of PRMT1 in almost all cancer types, including GC, than in the corresponding normal tissues (**Fig. S6A**). PRMT1 upregulation has been linked to numerous cancer types 67-70. However, the specific role of PRMT1 and c-Fos in GC remains to be elucidated. To expand the knowledge of the role that PRMT1 plays in GC pathogenesis, an RNA sequencing (RNA-seq) dataset from TCGA, including data from the Genotype-Tissue Expression (GTEx) database, and a number of human GC tissue microarray datasets were analyzed. PRMT1 expression was observed to be higher in stomach cancer tissues in GSE62254 (**Fig. 6A**), GSE26899 (**Fig. 6B**), GSE54129 (**Fig. 6C**), and GSE79973 (**Fig. 6D**) than in normal gastric tissues, normal samples in GTEx, or normal samples in TCGA (**Fig. 6E**), confirming that PRMT1 expression is high in patients with GC. Further profiling of PRMT1 expression was completed to characterize its expression in patients with various subtypes of GC. Intestinal-type GC categorized by Lauren classification showed significantly higher expression of PRMT1 mRNA than diffuse-type GC (**Fig. S6B**), suggesting that the expression of PRMT1 is more strictly associated with well-differentiated gastric tumor types than with poorly differentiated types. Importantly, the overall survival probability was compared between the groups of GC patients with low and high PRMT1 expression using Kaplan‒Meier Plotter (www.kmplot.com) with Affymetrix input ID 1565016_at, corresponding to the PRMT1 gene. The outcomes displayed that patients showing high PRMT1 expression had a lower survival probability (**Fig. 6F** and **Fig. S6C**). Interestingly, the distinction between the low and high PRMT1 groups in terms of survival was more pronounced in cohorts of patients with intestinal-type GC (hazard ratio, 2.08) (**Fig. 6F**). In addition, Kyoto Encyclopedia of Genes and Genomes (KEGG) 2021 human pathway enrichment analysis of PRMT1-associated genes identified in our RNA-seq analysis indicated a significant regulatory role of PRMT1 in GC (**Fig. S6D**). Together, these data validated the potential role of PRMT1 as an oncogene in GC. Furthermore, GSEA of the RNA-seq data with PRMT1-knockdown MKN45 cells (shPRMT1) as a control and PRMT1-knockdown MKN45 cells with reconstitution of PRMT1 expression (recovery) (**Fig. S7B**) showed that PRMT1 is negatively associated with the regulation of autophagy genes (false discovery rate [FDR]-adjusted *P* = 0.009, normalized enrichment score [NES] = -1.36) (**Fig. 6G**). Moreover, positive correlations of PRMT1 with gene markers (FDR-adjusted *P* = 0.000, NES = 1.66) and FOS target genes (FDR-adjusted *P* = 0.030, NES = 1.43) were found in advanced GC (**Fig. 6H** and** I**). This suggests that the promoting effect of PRMT1on GC is related to c-Fos target genes and autophagy pathways. Among autophagy genes in the Reactome database, PRMT1 was negatively correlated with genes such as *ATG9A*, *DYNC1H1*, *EPAS1*, *PINK1*, *TSC1*, *TUBB6*, and *ULK1* in gastric tumor tissues (STAD-TCGA) (**Fig. 6J** and** S6E**) and positively correlated with *HMMR*, *ESCO2*, *CHAC2*, and *NUDT6* (STAD-TCGA) (**Fig. 6K** and** S6F**). Of interest, these genes are FOS target genes, based on analysis of chromatin immunoprecipitation sequencing datasets from the ENCODE Transcription Factor Targets database (ncbi.nlm.nih.gov/gene/2353). Thus, we focused on how PRMT1-methylated c-Fos contributes to gastric tumorigenesis.

### PRMT1-methylated c-Fos supports gastric tumorigenesis

**Figure 7:**
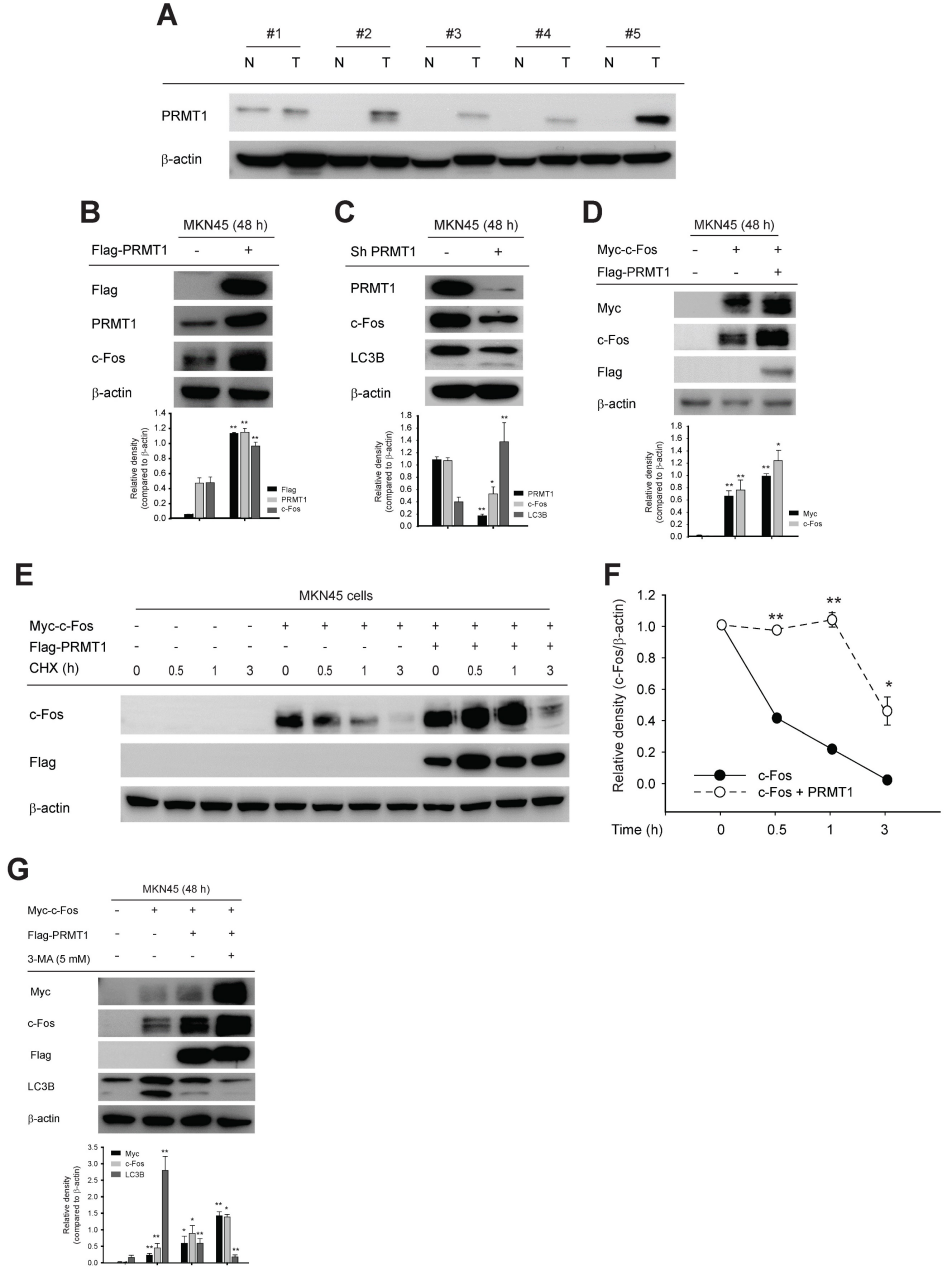
PRMT1 expression in GC tissues and the role of PRMT1 in c-Fos protein expression in GC cell lines. (A) PRMT1 expression in paired normal stomach tissues (N) and GC samples (T) from human patients was evaluated by immunoblot analysis. (B and C) c-Fos level was evaluated in MKN45 cells with overexpression (B) or knockdown (C) of PRMT1 together with LC3B levels. (D) Myc-c-Fos- and Myc-c-Fos/Flag-PRMT1-overexpressing MKN45 cells were harvested and used for immunoblot analysis of Myc, c-Fos, and Flag levels. (E) MKN45 cells overexpressed Myc-c-Fos and Flag-PRMT1. CHX was further applied for the indicated times, and the translational levels of c-Fos and Flag were measured in whole-cell lysates by immunoblotting; then, (F) the relative density of the c-Fos protein band was measured by ImageJ. (G) Myc-c-Fos and Flag-PRMT1 were cotransfected into MKN45 cells for 2 days, and 3-MA (5 mM) was added. The translational levels of Myc, c-Fos, Flag, and LC3B were measured by immunoblotting.

We used cell lines to examine the molecular relationship between PRMT1 and c-Fos in the milieu of GC. Consistent with c-Fos protein, PRMT1 protein level was higher in human GC cells (MKN1 and MKN45) than in a normal gastric cell line (HFE-145) (**Fig. S7A**). In paired normal gastric tissues and GC samples from human patients, higher PRMT1 expression in GC samples was confirmed (**Fig. 7A**). In addition, c-Fos expression was increased and decreased with overexpression and knockdown of PRMT1, respectively, in both MKN45 and MKN1 cells, as revealed by immunoblotting (**Fig. 7B**,** C**,** S7C**, and** S7D**). These outcomes indicate that the c-Fos protein level can be positively affected by the PRMT1 expression level. Furthermore, in line with findings in HEK293T cells (**Fig. 3A**), the c-Fos expression level was higher in c-Fos/PRMT1-cotransfected MKN45 cells compared with c-Fos-control-transfected MKN45 cells (**Fig. 7D**). We hypothesized that PRMT1 promotes GC by regulating c-Fos stability. To confirm this hypothesis, we tested c-Fos degradation using a CHX chase assay in MKN45 cells. As appeared in **Fig. 7E** and** F**, the c-Fos level decreased over the 3-h period but remained higher in the c-Fos/PRMT1-cotransfected group than in the c-Fos-transfected group. Moreover, consistent with data shown earlier (**Fig. 3D** and** 4B**), 3-MA treatment enhanced the PRMT1-mediated enhancement of c-Fos, and both protein-translational pattern and stability of c-Fos were elevated by PRMT1 transfection (**Fig. 7G**). This might be the result of inhibition of LC3B.

**Figure 8:**
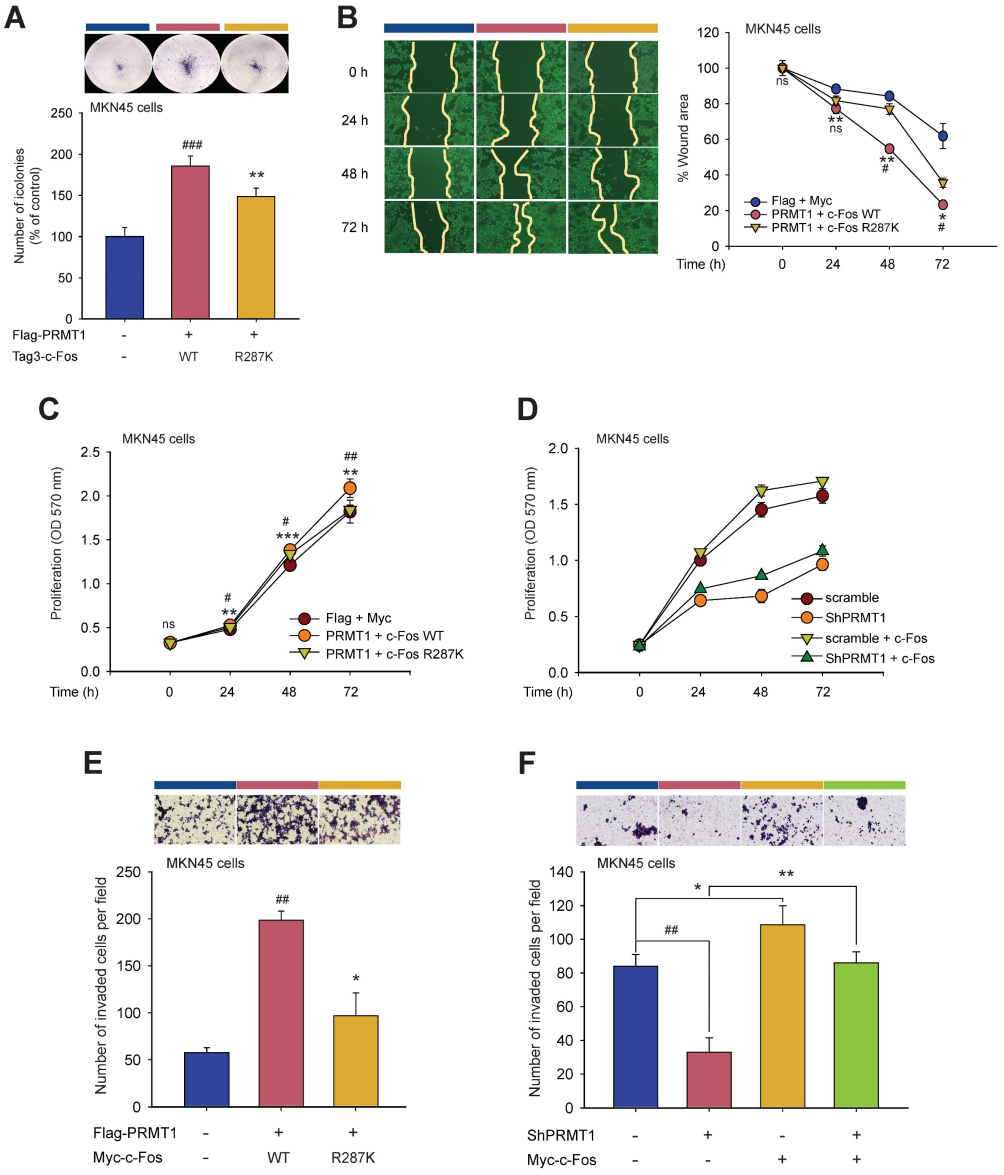

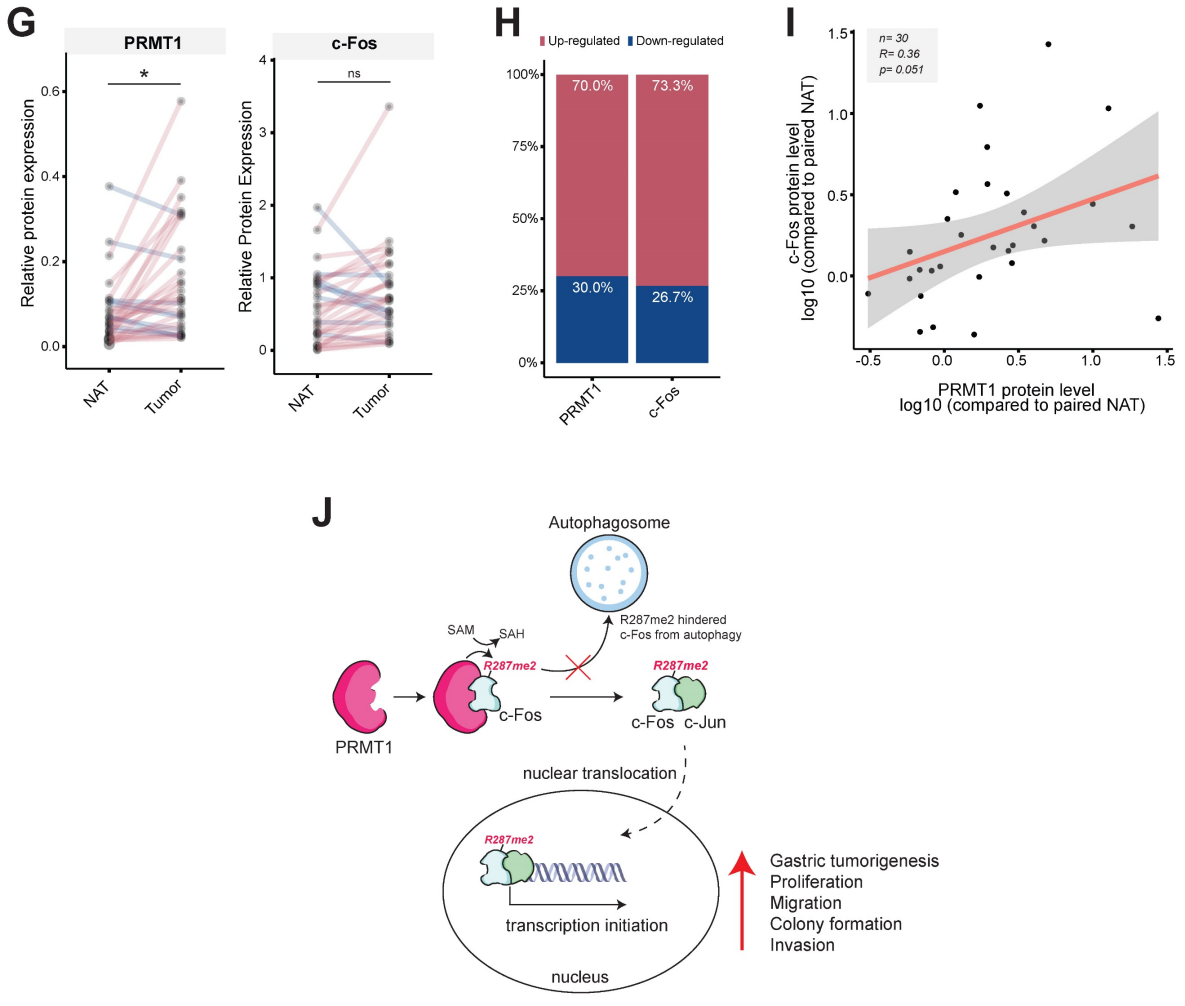
PRMT1-mediated methylation of c-Fos at R287 supports gastric tumorigenesis. Cellular responses were examined in c-Fos WT-overexpressing or R287K mutant (nonmethylated)-overexpressing MKN45 cells. (A) A clonogenic assay was performed in a six-well plate (1 x 10^3^ cells/well) by incubation for ≥10 days. The culture medium was taken away, the cells were fixed, stained, and imaged. (B) A wound healing effect was utilized to evaluate the migrative capability of MKN45 cells. Cells were imaged after indicated times, and the wound closure rate was determined by ImageJ. (C and D) Proliferation was evaluated at the indicated days by an MTT assay in (C) WT c-Fos- or nonmethylated c-Fos-cotransfected MKN45 cells and (D) c-Fos-overexpressing MKN45 cells expressing Scramble or shPRMT1. (E and F) An invasion assay was performed with c-Fos WT/Flag-PRMT1 or c-Fos R287K/Flag-PRMT1-cotransfected MKN45 cells (E) or c-Fos-overexpressing MKN45 cells expressing Scramble or ShPRMT1 (F). Invaded cells were tinted with hematoxylin and eosin Y solution, imaged using a camera connected to a microscope, and counted across three random areas of each well. (G) Pairwise analysis and (H) bar plot of the relative protein expression of PRMT1 and c-Fos in paired normal gastric tissue and GC samples from human patients. The relative expression of PRMT1 and c-Fos in tissues compared to MKN45 cells (control) was measured and normalized to that of β-actin determined by immunoblotting. Statistical significance was evaluated using the paired *t* test. (I) The correlation between PRMT1 and c-Fos expression in paired normal gastric tissue and GC samples from human patients was evaluated by immunoblotting and plotted. The correlation coefficient (R) between PRMT1 and c-Fos expression was calculated using Pearson correlation analysis. (J) A schematic diagram showing how PRMT1 regulates c-Fos activity by methylation. PRMT1 methylates the R287 residue of c-Fos and protects c-Fos from autophagic degradation. The increased protein stability of c-Fos leads to an increase in the functionality of AP-1 to foster gastric tumorigenesis and tumorigenic behaviors in GC cell lines.

To determine whether PRMT1-mediated methylation of c-Fos fosters gastric tumorigenesis, the functional role of c-Fos was examined by comparing cellular responses in PRMT1-overexpressing and PRMT1-knockdown cells cotransfected with WT c-Fos or the c-Fos R287K mutant (nonmethylated form). Overexpression and silencing of PRMT1 were performed in MKN45 cells, and the PRMT1 level was confirmed through immunoblotting (**Fig. S8A and S8B**). In combination with PRMT1 overexpression, c-Fos WT overexpression promoted tumorigenic responses, including colony formation (**Fig. 8A**), migration (**Fig. 8B, S8C,** and** S8D**), accelerated cell growth (**Fig. 8C** and** D**), and invasion (**Fig. 8E** and** F**). On the other hand, overexpression of nonmethylated c-Fos significantly suppressed these responses compared to those in WT c-Fos-expressing MKN45 cells. Besides, knockdown of PRMT1 repressed cellular growth and invasion, but c-Fos overexpression restored these responses (**Fig. 8D** and** F**). Hence, the data demonstrated that PRMT1-methylated c-Fos plays an important role in gastric tumorigenesis.

Moreover, using immunoblot analysis of samples in paired normal gastric tissues and GC samples from human patients, the correlation between the PRMT1 and c-Fos protein levels was evaluated (**Fig. S8E**). Pairwise analysis of PRMT1 and c-Fos protein expression, bar plot generation, and correlation analysis were performed to visualize differences in the normalized PRMT1 and c-Fos levels between NATs and GC samples. PRMT1 protein expression was significantly higher in GC samples than in NATs from human patients (n = 30, *P* = 0.010), with 70% of samples showing an increased PRMT1 level, while c-Fos was expressed in tumor tissue in approximately 73.3% of patients. Our findings suggest that the PRMT1 and c-Fos proteins are both highly expressed in GC samples compared to NATs from human patients (**Fig. 8G** and** H)**. Moreover, correlation analysis showed a moderate positive correlation of PRMT1 and c-Fos expression in GC samples compared to NATs (R = 0.36) (**Fig. 8I**), suggesting that PRMT1 appears to have a synergistic effect on c-Fos expression in patients with GC.

## Discussion

In our present research, the regulatory function of PRMT1 on the activation of c-Fos was delineated. Methylation of c-Fos protects it from autophagic degradation, which contributes to gastric tumorigenesis. We first observed a direct interaction between c-Fos and PRMT1, and that interaction synergistically enhanced c-Fos-mediated AP-1 activity. This phenomenon was dependent on PRMT1-mediated c-Fos methylation. Moreover, our study revealed c-Fos R287 as a new substrate of PRMT1 for arginine methylation. In addition, PRMT1 protected the c-Fos protein from autophagic degradation, enhanced c-Fos protein stability, and enhanced c-Fos activity, impacting human gastric tumorigenesis. Hence, our study established an important function of PRMT1 in c-Fos/AP-1 regulation, and targeted inhibition of this axis might be another approach for treating GC.

PRMT1 is a methyltransferase that regulates various cellular processes via methylation or another mechanism. AP-1 can contain various subunits (c-Fos, ATF, and c-Jun family members), and its activation is induced by various ligands. As a result, AP-1 mediates diverse cellular reactions, including differentiation, proliferation, and tumorigenesis. Other studies have demonstrated that posttranslational modifications such as SUMOylation, ubiquitination, and phosphorylation can regulate c-Fos activity.

c-Fos is commonly phosphorylated at several sites by p70S6K, MAPKs, protein kinases A and C, or 90-kDa ribosomal S6 kinase 71,72, c-Fos complexes on target gene promoters can be SUMOylated to promote transcription 20. However, c-Fos protein methylation is not well understood. Some reports have examined the ability of c-Fos DNA methylation to regulate its gene expression 73-75. Our data showed that PRMT1 specifically increases c-Fos-mediated AP-1 luciferase activity. We detected the interaction of c-Fos and PRMT1 and identified an increased level of methylated arginine on c-Fos. We discovered four arginine-methylated sites on the c-Fos protein, among which PRMT1-methylated c-Fos R287 appears to specifically regulate c-Fos protein stability. It was evident that the c-Fos transcription factor is methylated and expression levels of AP-1-regulated genes are altered via PRMT4. However, the methylated sites of c-Fos have not been identified 28. In summary, we demonstrated that c-Fos is posttranslationally methylated to regulate its stability and activity and identified the methylated arginine residues in c-Fos.

c-Fos proteins are short-lived proteins whose expression is tightly regulated by proteolytic pathways 76-79. c-Fos can be degraded by proteasomes via ubiquitin-dependent or ubiquitin-independent pathways. c-Fos degradation is differentially regulated based on the involvement of various destabilizers (kinases, enzymes, or c-Jun) 80,81. For example, ERK5 phosphorylation simultaneously regulates c-Fos nuclear translocation and stability 82. The interaction between c-Fos and NAD(P)H:quinone oxidoreductase 1 prevents c-Fos degradation through a ubiquitin-independent proteasomal pathway 77. Previous reports have shown that c-Fos can undergo proteasomal degradation. We demonstrated here that c-Fos degradation is activated via autophagy, which is an important intracellular degradation process mediated by lysosomes that is designed to reutilize cellular components. We found that c-Fos was located in autophagosomes and that knockdown of PRMT1 increased the protein level of the autophagic degradation marker LC3B. Autophagy is usually triggered by nutrient starvation 83,84, and 48 h was a sufficient length of time for induction of starvation. This indicates that PRMT1 plays a pivotal role in nutrient starvation by modulating c-Fos degradation. Although further studies are needed to understand how PRMT1-methylated c-Fos is protected from degradation, our research documents a new posttranslational mechanism that regulates c-Fos stability and activity.

Previous studies have demonstrated that transcription factors can be modulated by autophagic degradation. PU.1 undergoes autophagic degradation dependent on p62 (a selective autophagy adaptor) to suppress T helper 9 (Th9) cell differentiation 85. Transcriptional adjustment by hypoxia-inducible factor-1 (HIF-1) was shown to be modulated by autophagy 86. HIF-1α, a major subunit of HIF-1, is targeted by chaperone-mediated autophagy, resulting in altered expression of HIF-1 target genes. Thus, transcription factors could be regulated by degradation pathways (especially autophagy). Similar to these studies, our study shows that c-Fos-mediated AP1 activity is regulated by autophagic degradation of c-Fos.

The pathophysiological role of c-Fos is deeply studied, especially because c-Fos is a proto-oncogene that plays diverse roles in cells 25,26,28,87. In addition, various types of human cancers have been reported to exhibit PRMT1 upregulation 8,11,53,55. However, the roles and underlying mechanisms of PRMT1 and c-Fos in GC are not clearly understood. Here, we found upregulation of PRMT1 in patient tissues and established human gastric cancer cells. Notably, an intensive mRNA expression level of PRMT1 has been suggested to be an unfavorable marker in GC. Many biological processes, including cancer progression, involve arginine methylation of various substrate proteins 70. Interestingly, our research revealed that c-Fos expression and activity are regulated by PRMT1. Our findings demonstrate that arginine methylation of c-Fos at R287 increases the stability of the c-Fos protein and protects it from autophagic degradation.

This study also elucidates a pro-oncogenic function for PRMT1 in the regulation of gastric tumorigenesis. Analysis of PRMT1 gene expression was conducted in multiple GC cohorts, and the results confirmed the upregulation of PRMT1 in GC tissues in comparison with the NATs. Furthermore, enrichment analysis verified that PRMT1 positively and significantly regulates GC progression. These results support a previous finding about the important roles of PRMT1 in the progression of GC 31. Mechanistically, by increasing the stability of c-Fos, PRMT1-mediated c-Fos methylation accelerates the growth and enhances the invasion and migration of human GC cells. Conversely, the R287K mutation at the arginine methylation site of c-Fos reduced its stability and attenuated these tumorigenic phenotypes. In addition, the PRMT1-c-Fos signaling axis in GC was revealed by correlation analysis of PRMT1 and c-Fos protein levels in the Ajou cohort, indicating that the interaction of PRMT1 and c-Fos contributes to GC. Therefore, combined inhibition of c-Fos and PRMT1 enzymatic activity could inhibit tumorigenesis.

## Conclusions

In conclusion, our study suggests that the regulatory mechanism of c-Fos/AP-1 activity could be altered by PRMT1 (**Fig. 8J**). PRMT1 is a specific regulator of c-Fos and protects it from autophagic degradation by regulating its methylation. Notably, the correlation in the levels of these two proteins greatly contributes to an important role in gastric tumorigenic responses, and specific targeting of PRMT1-catalyzed arginine methylation of c-Fos could become a potential new therapeutic strategy for GC.

## Supplementary Material

Supplementary methods and figures.

## Figures and Tables

**Table 1:** List of primers used to generate the c-Fos mutant constructs.

Target		Sequence (5' to 3')
R108K	F	cgctggggcttactccaaggctggcgtt
	R	aacgccagccttggagtaagccccagcg
R201K	F	catcctggcagctcacaaacctgcctgcaagatc
	R	gatcttgcaggcaggtttgtgagctgccaggatg
R279K	F	ctgttcccagcatcatccaagcccagtggc
	R	gccactgggcttggatgatgctgggaacag
R287K	F	tggctctgagacagccaagtccgtgccagacatgg
	R	ccatgtctggcacggacttggctgtctcagagcca
R287F	F	ggctctgagacagccttctccgtgccagacat
	R	atgtctggcacggagaaggctgtctcagagcc

**Table 2:** Protein substrates interacting with the c-Fos protein.

Putative substrate	Accession	MW (kDa)	Subcellular location
Protein arginine N-methyltransferase 1	Q99873	41.5	Nucleus, Cytoplasm, Cytosol
Protein arginine N-methyltransferase 5	O14744	72.6	Nucleus, Cytoplasm, Cytosol, Golgi apparatus, Chromosome
Histone-lysine N-methyltransferase SETD2	Q9BYW2	287.4	Nucleus, Chromosome
Histone-lysine N-methyltransferase NSD2	O96028-1	152.2	Nucleus, Chromosome
Probable methyltransferase-like protein 15	A6NJ78	46.1	Mitochondrion
Histone-lysine N-methyltransferase SETD1A	O15047	185.9	Nucleus, Chromosome
Histone-lysine N-methyltransferase SMYD3	Q9H7B4-1	49.1	Nucleus, Cytoplasm, Cytosol
Methyltransferase-like protein 13	Q8N6R0-5	78.7	-
Histone-lysine N-methyltransferase EHMT2	Q96KQ7	132.3	Nucleus, Chromosome
Histone-lysine N-methyltransferase NSD3	Q9BZ95	161.5	Nucleus, Chromosome
Protein arginine N-methyltransferase 3	O60678	59.8	Cytoplasm

## Abbreviations

PRMT1: Protein arginine methyltransferase 1
AP-1: Activator protein-1
MAPKs: Mitogen-associated protein kinases
GC: Gastric cancer
WT: Wild-type
3-MA: 3-Methyladenine
CHX: Cycloheximide
NES: Normalized enrichment score
NATs: Normal adjacent tissues
GSEA: Gene set enrichment analysis
GEO: Gene Expression Omnibus
ShRNA: Short hairpin RNA
MS: Mass spectrometry
IP: Immunoprecipitation
RT‒PCR: Real-time polymerase chain reaction
