# Supplementary material for: Protection of c-Fos from autophagic degradation by PRMT1-mediated methylation fosters gastric tumorigenesis
Source: Int J Biol Sci. 2023 Jul 15;19(12):3640–60. doi: 10.7150/ijbs.85126 (PMC10411464; doi:10.7150/ijbs.85126)
Supplement: Supplementary file 1 — Supplementary methods and figures. [file ijbsv19p3640s1.pdf]

Supplementary Materials

Protection of c-Fos from autophagic degradation by PRMT1-mediated methylation fosters gastric tumorigenesis

Eunji Kim [1,2,+], Laily Rahmawati [1,3,+], Nur Aziz [1,4,+], Han Gyung Kim [1], Ji Hye Kim [1], Kyung-Hee Kim [5], Byong Chul Yoo [6], Narayana Parameswaran [7], Jong-Sun Kang [8], Hoon Hur [9], Balachandran Manavalan [1,*], Jongsung Lee [1,*], and Jae Youl Cho [1,*]

[1]Department of Integrative Biotechnology and Biomedical Institute for Convergence at SKKU (BICS), Sungkyunkwan University, Suwon 16419, Republic of Korea.
[2]R&D Center, Yungjin Pharmaceutical Co, Suwon 16229, Republic of Korea.
[3]Emergency Department, Hermina Hospital Tangkubanprahu, Malang 65119, Indonesia.
[4]Pharmacy Program, Faculty of Science and Engineering, Universitas Ma Chung, Malang 65151, Indonesia.
[5]Proteomic Analysis Team, Research Institute, National Cancer Center, Goyang 10408, Republic of Korea.
[6]Division of Translational Science, Research Institute, National Cancer Center, Goyang 10408, Republic of Korea.
[7]Department of Physiology and Division of Pathology, Michigan State University, East Lansing, Michigan 48824, USA.
[8]Department of Molecular Cell Biology, Single Cell Network Research Center, Sungkyunkwan University School of Medicine, Suwon 16419, Republic of Korea.
[9]Department of Surgery, Department of Biomedical Sciences, Ajou University School of Medicine, Suwon 16499, Republic of Korea.

[+]These authors contributed equally to this work

[*]Corresponding authors: Jae Youl Cho; jaecho@skku.edu, Jongsung Lee: bioneer@skku.edu, and Balachandran Manavalan: bala2022@skku.edu

Materials and Methods

Chemicals and antibodies

MG132, 3-methyladenine (3-MA), cycloheximide (CHX), polyethylenimine (PEI), and digitonin (1211877-36-9, 5142-23, C7698, 9002-98-6, and 11024-24-1) were purchased from Sigma-Aldrich (St. Louis, MO, USA). AP-1 response elements containing luciferase constructs were purchased from Promega Corporation (Madison, WI, USA). Plasmids (Myc–c-Fos, Flag–c-Fos, Flag-PRMT1, and EGFP-PRMT5) were cloned, and EGFP-PRMT1 and -PRMT3 plasmids were kind gifts from Prof. Kim (Sookmyung Women's University), while the pLKO.1 vector was obtained from Addgene (10878), having been deposited by David Root. Proteinase K (KB-0111) was acquired from Bioneer (Daejeon, Korea). The following primary antibodies were used: LC3B, mme-R, adme-R, Myc, and Flag (3868, 8015, 13522, 2276, and 8146; Cell Signaling Technology, Danvers, MA, USA). Antibodies against c-Fos, β-actin, and GFP (sc-166940, sc-47778, and sc-9996; Santa Cruz Biotechnology, Dallas, TX, USA) were secured, and staining antibodies included anti-mouse Alexa 405 (A-31553) and anti-rabbit Alexa 568 (A-11011) (Invitrogen, Carlsbad, CA, USA).

Cell culture

HEK293T (CRL-1573; ATCC, Manassas, VA, USA), MKN45, and MKN1 cells (80103 and 80101; KCLB, Seoul, Korea) were cultured in DMEM or RPMI1640 (SH30243.01 or SH30027.01; Hyclone Laboratories, Logan, UT, USA) with 10% heat-inactivated fetal bovine serum (FBS) (16000-044; Gibco Laboratories, Gaithersburg, MD, USA) and 1% penicillin–streptomycin (SH40003.01; Cytiva, Marlborough, MA, USA) in a 5% CO_2 humidified incubator at 37°C as previously reported [33].

Constructs and mutagenesis

Constructs for c-Fos and PRMT5 were generated by PCR using HEK293T cDNA as a template. The PRMT1 DN mutant (63VLD64 to 63AAA65) was generated using the QuickChange site-directed mutagenesis system (Stratagene, San Diego, CA, USA). c-Fos methylated point mutants (R108K, R201K, R279K, R287K, and R287F) were generated using the same method. Primers used for generation of site-directed mutants are listed in **Table 1**.

Lentivirus-mediated knockdown (short hairpin RNA (shRNA)

The shRNA coding sequences against PRMT1-containing plasmids were cloned according to Addgene protocols (www.addgene.org). Non-targeting scrambled shRNA (TCCTAAGGTTAAGTCGCCCTCG) sequences and PRMT1 shRNA sequences (CCGGCAGTACAAAGACTACAA) were cloned into the pLKO.1 vector. Lentivirus was produced by transfection of transient HEK293T cells. Generated viruses were injected into cells, and either shScramble- or shPRMT1-infected cells were selected by puromycin treatment. Knockdown level of PRMT1 was confirmed by immunoblotting.

Transfection of DNA and the luciferase reporter gene activity assay

HEK293T cells were transfected with empty vectors or the indicated plasmids (c-Fos, PRMT1, PRMT3, or PRMT5) (0.25 μg/well), Luc-constructs (0.25 μg/well), and β-galactosidase (0.1 μg/well) using PEI in a 24-well plate as previously reported [34]. After 24 h, cell media was changed to FBS-containing media or compound and treated for an additional 24 h. The transfected cells were lysed to determine luciferase activity using the luciferase assay system (E1500; Promega Corporation). Luciferase activity was normalized to that of β-galactosidase.

Preparation of whole-cell lysates, tissues, and immunoblotting

A total of 30 pairs of GC patient tumor tissues and normal adjacent tissues (NATs) was obtained from patients who underwent surgical resection of GC at Ajou University Hospital. Specimens were collected at Ajou Human Bio-Resource Bank and frozen at −80°C until use. Written informed consent for the usage and storage of specimens was received from all patients. The present study was conducted in accordance with the ethics code of the World Medical Association (Declaration of Helsinki) and was approved by the Institutional Review Board of Ajou University Hospital (AJIRB-BMRKSP-19-059) as previously reported [30,33]. The GC patient tumor tissues and NATs specimens were ground with liquid nitrogen, while the cell lines were washed with phosphate-buffered saline (PBS) (B2814; Samchun Pure Chemical, **Pyeongtaek, Korea**), collected, and centrifuged at 12,000 rpm for 5 min at 4°C. They then were lysed with buffer (20 mM Tris-HCl [pH: 7.4], 2 mM of ethylenediaminetetraacetic acid [EDTA], 2 mM of ethyleneglycotetraacetic acid [EGTA], 1 mM of DTT, 50 mM of β-glycerol phosphate, 0.1 mM of sodium vanadate, 1.6 mM of pervanadate, 1% Triton X-100, 10% glycerol, 10 μg/mL of aprotinin, 10 μg/mL of leupeptin, 10 μg/mL of pepstatin, 1 mM of benzamide, and 50 μM of PMSF). Protein lysates were pelleted via centrifugation (12,000 rpm, 5 min, 4°C), and the supernatant was used for western blotting with antibodies against Myc, Flag, GFP, total c-Fos, LC3B, mme-R, adme-R, and β-actin [35].

Immunoprecipitation

Cell lysates containing equal amounts of protein (1,000 μg) were prepared, and samples were incubated with 5 μL of antibodies overnight at 4°C. Immune complexes were mixed with 40 μL of protein A- or G-coupled Sepharose beads (50% v/v) and rotated for 4 h at 4°C. Boiled immune complexes were immunoblotted, and the protein levels were determined as previously reported [36-38].

Immunofluorescence and image analysis

HEK293T cells were plated on a glass coverslip overnight and transfected with Myc–c-Fos and EGFP-PRMT1 for 48 h. Cells were fixed in 3.7% paraformaldehyde in PBS and then permeabilized with 1% Triton X-100 in PBS at room temperature. After washing, cells were treated with 1% bovine serum albumin in PBS to minimize nonspecific staining. For staining, cells were incubated with anti-Myc, anti-Flag, or anti–adme-R (1:1000 dilution) for 1 h at room temperature. Secondary antibodies were conjugated with Alexa Fluor (1:000 dilution) for 1 h and then with Hoechst staining solution (1:1000 dilution) for 20 min at room temperature. DAPI was used for DNA counterstaining [39]. The coverslips were mounted on slide glasses using fluorescent mounting medium (DakoCytomation, Carpentaria, CA, USA). A confocal laser-scanning microscope (LSM 700; Zeiss, Jena, Germany) equipped with a C-Apochromat 63×/1.2 water immersion objective was used to acquire images [40,41].

mRNA analysis by semi-quantitative RT-PCR or quantitative real-time PCR

To examine the mRNA expression level of c-Fos, HEK293T cells were transfected with Myc–c-Fos and EGFP-PRMT1. The total RNA from the cells was isolated with TRI reagent (TR118; Molecular Research Center, Cincinnati, OH, USA) according to the manufacturer's instructions. The total RNA was stored at −70°C until use. Semi-quantitative RT-PCR was performed as previously reported [42]. The mRNA quantification was conducted via real-time RT-PCR with SYBR Premix according to the manufacturer's instructions (PCRBIOSYSTEMS, London, UK) using a real-time thermal cycler (Bio-Rad Laboratories, Hercules, CA, USA) as previously reported [43,44].

Protease protection assay

The protein level in proteasomes was evaluated by a protease protection assay according to a previous report [45]. c-Fos or c-Fos/PRMT1 plasmids were transfected (3 samples/group) for the indicated time, and the reaction was stopped by adding cold PBS. Cells were harvested and washed twice with PBS. Next, 6.5 μg/mL of digitonin solution (100 mM of potassium phosphate [pH: 6.7], 5 mM of $MgCl_2$, and 250 mM of sucrose) was added to cells (2 samples/group) that then were washed with cold PBS. Cells were incubated with 1 μg/mL of proteinase K for 10 min with or without 0.1% Triton X-100, which dissolved all membranes. Buffer was removed, and sample buffer was added for protein analysis. β-actin was used as a protein control.

LC-MS/MS spectrometry

Myc–c-Fos–transfected cells were collected and immunoprecipitated with Myc. Immunoprecipitated samples were separated by sodium dodecyl sulfate–polyacrylamide gel electrophoresis and stained with Coomassie blue dye. The samples were subjected to tryptic digestion at 37°C overnight; followed by lyophilization, reconstitution, and fractionation; and then analyzed by strong cation exchange (SCX) liquid chromatography (LC) and mass spectrometry.

QuantSeq 3' mRNA sequencing

PRMT1-knockdown MKN45 cells and MKN45 cells recovered from PRMT1-knockdown were harvested, and total RNAs were isolated with TRIzol reagent. RNA sequencing and data analysis were performed by E-Biogen Inc. (Seoul, Korea). Library construction was performed using the QuantSeq 3' mRNA-Seq Library Prep Kit (Lexogen, Inc., Greenland, NH, USA) according to the manufacturer's protocol. High-throughput sequencing was performed as single-end 75 sequencings using a NextSeq 500 (Illumina, Inc., San Diego, CA, USA). The Excel-based Differentially Expressed Gene Analysis (ExDEGA) GraphicPlus v2.0 software provided by EBiogen Inc. was used for filtering DEGs (fold change > 2). GSEA of selected genes was conducted using GSEA software (http://software.broadinstitute.org/gsea/index.jsp) [33].

Microarray and RNA-seq analysis from a publicly available database

GSE66229 [46], GSE26899 [46], GSE54129 (unpublished), and GSE79973 [47] containing gene-expression profiles of normal and gastric tumor tissues were retrieved from Gene Expression Omnibus (GEO) (https://www.ncbi.nlm.nih.gov/geo/). Unified STAD normal, STAD tumor, and healthy GTEx stomach tissues were downloaded from Schultz et al. [48]. Non-pairwise comparisons were performed in R version 4.2.1 (R Foundation for Statistical Computing, Vienna, Austria).

Multiple sequence alignments

The PRMT1 FASTA protein sequences of *Homo sapiens* (human), *Mus musculus* (mouse), *Rattus norvegicus* (rat), *Macaca mulatta* (Rhesus macaque), *Pan troglodytes* (chimpanzee), *Bos taurus* (bovine), *Canis lupus familiaris* (dog), *Gallus gallus* (chicken), *Danio rerio* (zebrafish), and *Xenopus tropicalis* (western clawed frog) were retrieved from the Uniprot database (www.uniprot.org). Multiple sequence alignment was performed using ClustalW with default parameters in Jalview 2.11.1.4.

Cell proliferation assay

PRMT1-overexpressing MKN45 cells or PRMT1-knockdown MKN45 cells were co-transfected with c-Fos–WT or mutants (1.5 × 10^3 cells/well). Cells were pre-incubated in 96-well plates (30096; SPL Life Sciences, Pocheon, Korea). After 12 h of incubation (0 days), 10 μL of MTT solution (10 mg/mL in PBS [pH: 7.4]) was added to the cell culture for 3 h at 37°C; then, 100 μL of stop solution (15% sodium dodecyl sulfate) was added to stop the reaction, followed by overnight incubation (37°C, 5% CO_2). The same steps were repeated after 24, 48, and 72 h. The absorbance was measured at 570 nm using a Synergy HT Multi-Mode Microplate Reader (BioTek Instruments GmbH, Bad Friedrichshall, Germany) as previously reported [30]. Data are shown as the mean ± standard deviation (SD).

Wound healing assay

MKN45 cells were transfected with empty vectors or the indicated plasmids (PRMT1 and c-Fos WT or c-Fos R287K) and seeded in a 12-well plate with 5.5 × 10^5 cells/well. Cells were incubated overnight at 37°C under 5% CO_2 and then scratched using sterile P200 pipette tips in a straight line in the center of the wells, which then received new media. Cells were photographed after 0, 24, 48, and 72 h, and the wound closure rate was determined by ImageJ analysis (U.S. National Institutes of Health, Bethesda, MD, USA). Data are shown as the mean ± SD.

Colony formation assay

MKN45 cells were transfected with empty vectors or the indicated plasmids (PRMT1 and c-Fos WT or c-Fos R287K) for 24 h using Lipofectamine 2000 (11668-019; Invitrogen). Cells were seeded (0.5 × 10^3 cells/well) in six-well plates. After 10 days of incubation (37°C, 5% CO_2), the colonies were fixed with 4% paraformaldehyde for 15 min and stained with 0.5% crystal violet (C3886; Sigma-Aldrich). The number of colonies was captured using a digital camera and counted using ImageJ. Data are shown as the mean ± SD.

Invasion assay

PRMT1-overexpressing MKN45 cells or PRMT1-knockdown MKN45 cells were co-transfected with c-Fos–WT or mutants for 24 h using Lipofectamine 2000. Cells were plated (5 × 10^4 cells/well) in an upper Transwell chamber with membrane-permeable polycarbonate filters coated with Matrigel (356237; BD Biosciences, San Diego, CA, USA) in Opti-MEM (11058021; Gibco Laboratories). The bottom chamber of the 24-well plate was filled with RPMI 1640 as a chemoattractant. After 24 h incubation (37°C, 5% CO_2), the upper chamber was removed, followed by fixation of cells on the lower side (invaded cells) using 4% paraformaldehyde. Invaded cells were stained with hematoxylin (ab220365; Abcam, Cambridge, US) and eosin y solution (HT110116; Sigma-Aldrich). The invaded cells were captured using a camera microscope and counted in three random fields per well. Data are shown as the mean ± SD.

Statistical analysis

Statistical analysis and data visualization were performed using SigmaPlot 11.0 (Systat Software, San Jose, CA, USA) or R (version 4.2.1). The results were analyzed by Student's *t* test or the Mann–Whitney *U* test. The survival curve was evaluated by Kaplan–Meier analysis. $P < 0.05$ was considered statistically significant.

Supplementary Figure legends

Figure S1. AP-1 activity after cotransfection under several conditions. (A) Diagram of strategy for detecting the immunoprecipitated c-Fos-bound proteins by LC–MS/MS. (B) c-Jun and PRMT1 were cotransfected into HEK293T cells for 48 h. c-Jun-mediated AP-1 luciferase activity was measured by a luminometer. (C) c-Fos and methyltransferases were cotransfected into HEK293T cells for 48 h. c-Fos-mediated AP-1 luciferase activity was measured by a luminometer. $^{##}P < 0.01$ versus the normal group and $^{**}P < 0.01$ versus the c-Jun, c-Fos alone, or c-Fos/PRMT1 cotransfection group.

Figure S2. Diagram of the wild-type (WT) and dominant negative (DN) forms of PRMT1. The VLD residues in the methyltransferase domain were replaced with AAA to generate the PRMT1 DN mutant.

Figure S3. Expression, activity, phosphorylation, nuclear translocation, and dimerization of c-Fos and c-Jun upon cotransfection of c-Fos, PRMT1, and MAPKs. (A and B) The expression level of c-Fos under c-Fos and PRMT1 cotransfection conditions was measured by RT–PCR (A) and real-time PCR (B). (C) c-Fos and PRMT1 or MAPKs (ERK2, JNK2, and p38) were cotransfected into HEK293T cells for 48 h. AP-1-mediated luciferase activity was measured by a luminometer. (D, E, and F) The levels of Myc, GFP, and Flag and the levels of phosphorylated c-Fos and MAPKs were measured by immunoblotting in whole-cell lysates (D and E) and the anti-Myc immunoprecipitate (F) of HEK293T cells transfected with c-Fos, c-Jun, or PRMT1. $^{##}P < 0.01$ versus the normal and $^{**}P < 0.01$ versus the c-Fos alone.

Figure S4. Autophagic degradation of c-Fos and autophagy-inducing activity of PRMT1. (A) HEK293T cells were transfected with Myc-c-Fos and EGFP-PRMT1 prior to treatment with the autophagy inducer rapamycin (1 μM) for an additional 24 h. AP-1-mediated luciferase activity was measured by a luminometer. (B) PRMT1 and LC3 I/II levels in whole-cell lysates of PRMT1-knockdown HEK293 cells were measured by immunoblotting.

Figure S5. Sequence alignment of c-Fos arginine methylation sites across species.

Figure S6. PRMT1 gene expression is increased in various types of cancers, including GC, and is associated with poor prognosis in GC. (A) The DiffExp module in TIMER was used to evaluate the expression of PRMT1 in various tumor vs. normal tissues in TCGA. (B) PRMT1 gene expression analysis in gastric cancer subtypes in the TCGA_STAD cohort based on the Lauren classification. (C) Survival was assessed based on PRMT1 expression in cohorts of patients with the diffuse subtype of gastric cancer using Kaplan–Meier Plotter (www.kmplot.com). (D) Kyoto Encyclopedia of Genes and Genomes 2021 pathway enrichment analysis of PRMT1-associated genes. (E and F) The correlation coefficient (R) calculated using Pearson correlation analysis and p values between PRMT1 expression and the expression of several genes are shown in Figures 6J and K.

Figure S7. PRMT1 regulates c-Fos expression in GC cell lines. (A) PRMT1 and c-Fos protein levels in normal gastric cells (HFE-145) and GC cell lines (MKN1 and MKN45) were measured using immunoblotting. (B) Knockdown and reconstitution efficiency in PRMT1-silenced MKN45 cells. (C and D) The c-Fos protein level was evaluated in MKN1 cells with overexpression (C) and knockdown (D) of PRMT1.

Figure S8. PRMT1-mediated methylation of c-Fos at R287 promotes gastric tumorigenesis. (A and B) MKN45 cells were transfected with Myc-c-Fos under PRMT1-knockdown conditions (A) and with WT c-Fos or nonmethylated c-Fos under PRMT1-overexpressing conditions (B). The cells were analyzed to assess responses. (C and D) A wound healing assay was utilized to evaluate the migration of MKN1 cells. Cells were photographed after 0, 24, and 48 h; then, the wound closure rate was determined by ImageJ. (E) Western blot analysis of PRMT1 and c-Fos protein expression levels in paired normal gastric (N) and GC (T) samples from human patients.

Figure S1

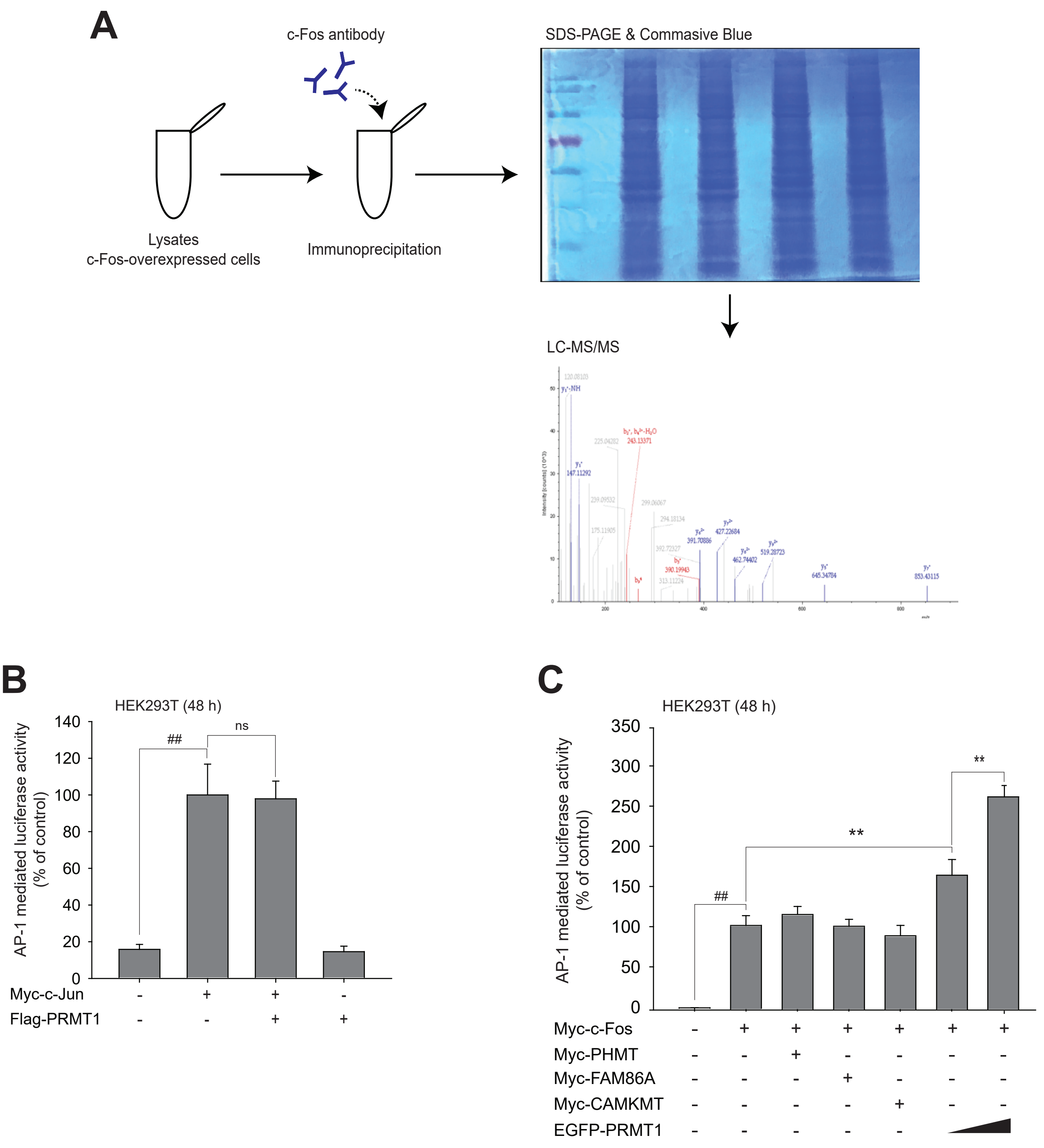

Figure S2

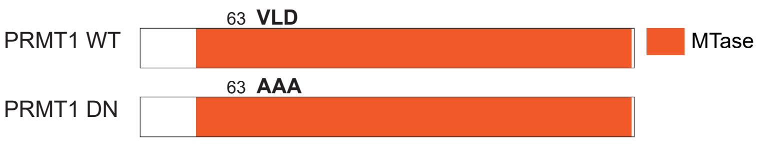

Figure S3

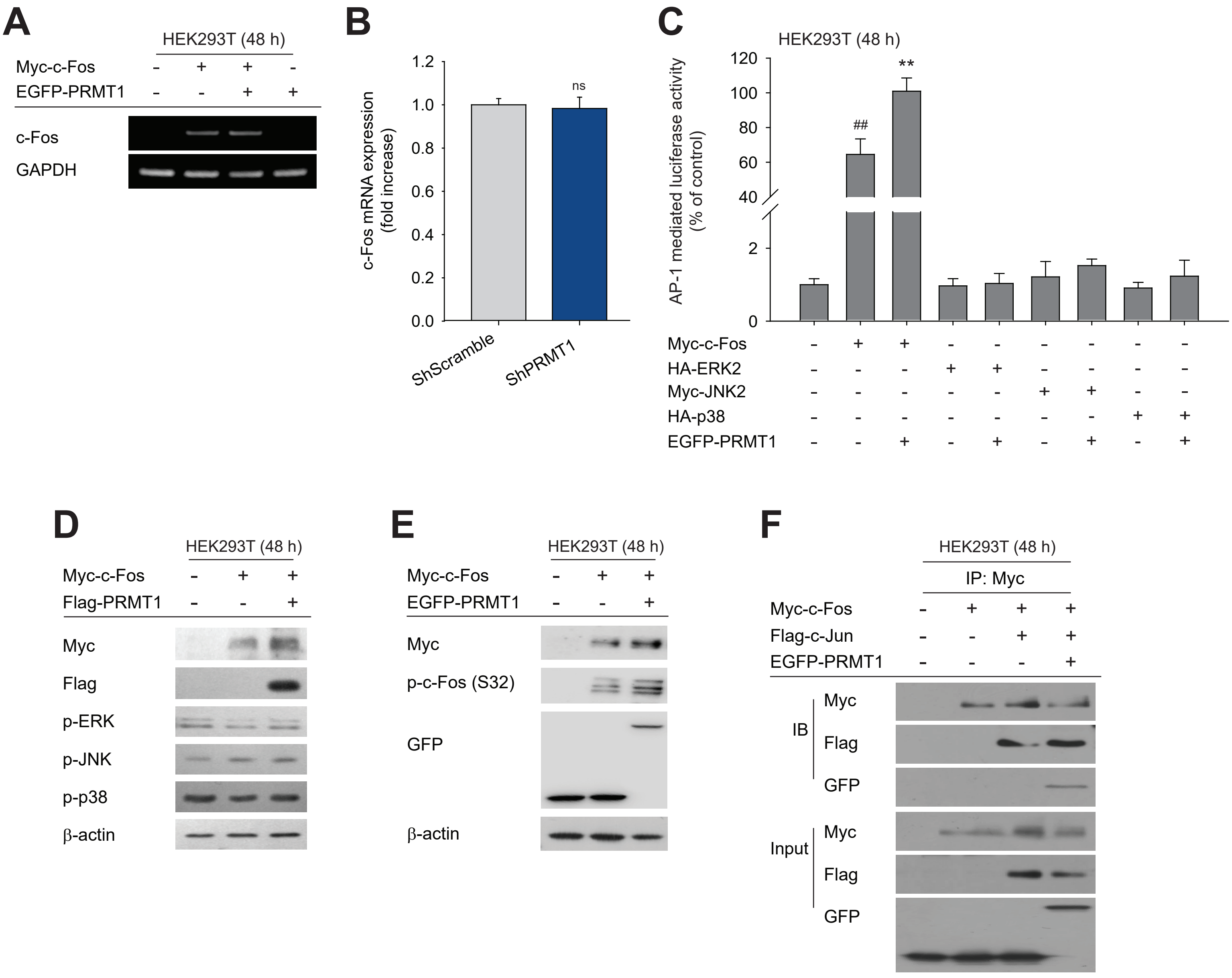

Figure S4

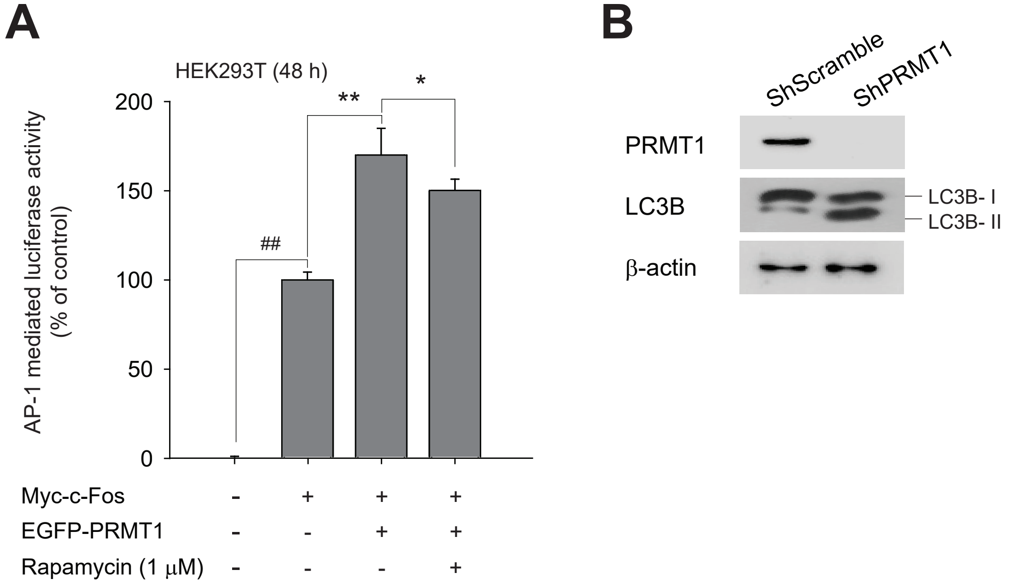

Figure S5

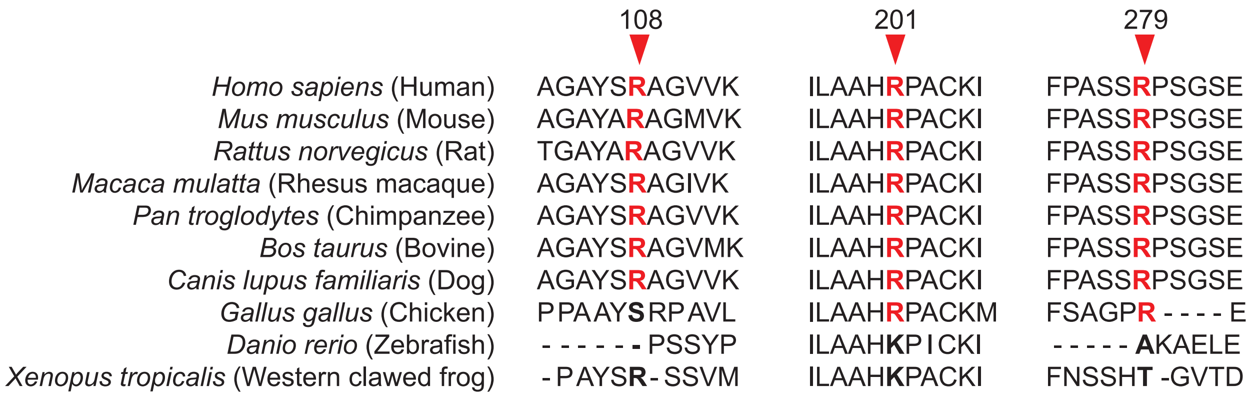

Figure S6

A

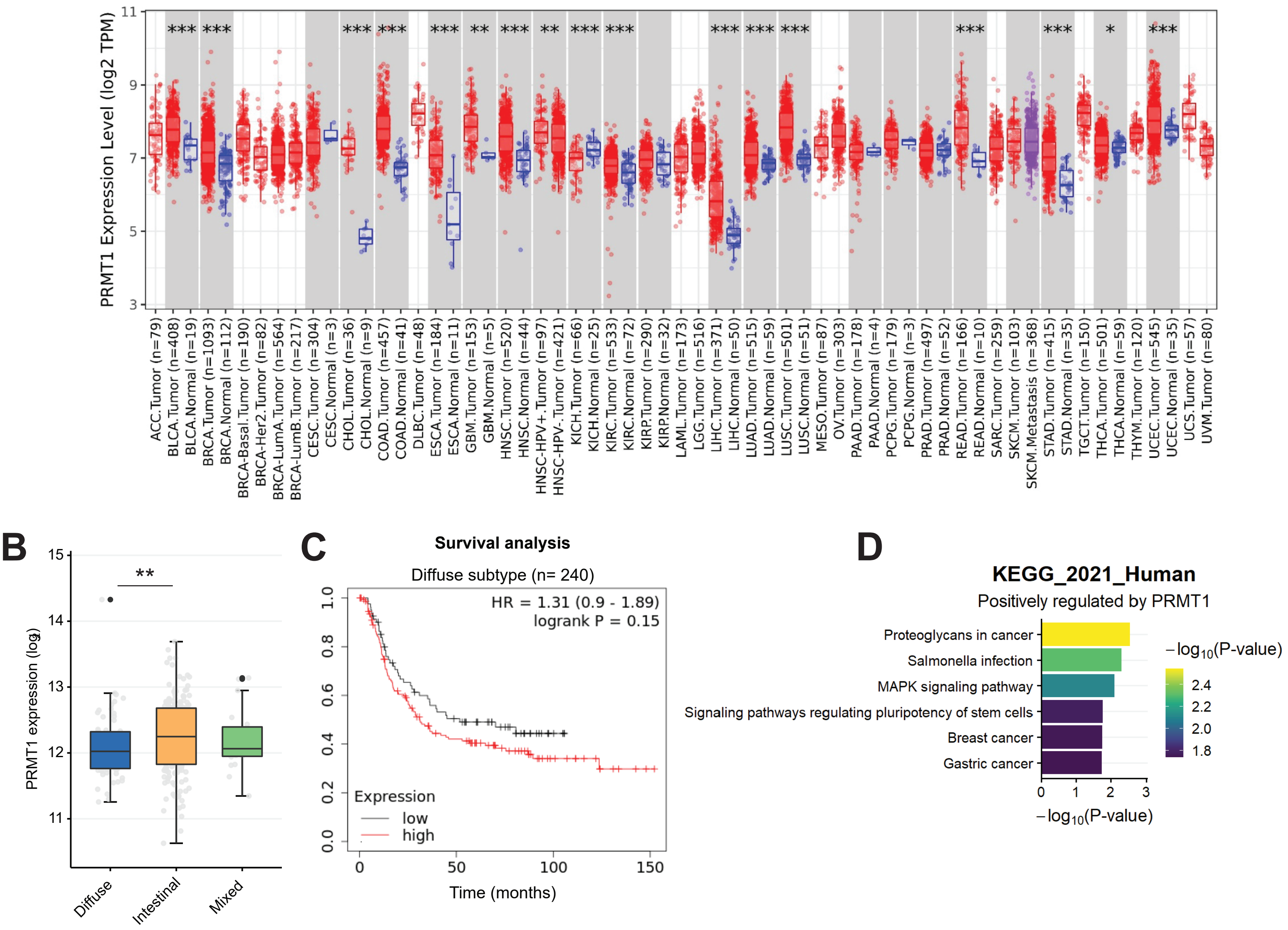

E

Parameter1	Parameter2	r	95% CI	t(412)	p
PRMT1	TUBB6	-0.22	[-0.31, -0.13]	-4.56	< .001***
PRMT1	PINK1	-0.32	[-0.41, -0.23]	-6.89	< .001***
PRMT1	EPAS1	-0.41	[-0.49, -0.33]	-9.17	< .001***
PRMT1	DYNC1H1	-0.23	[-0.32, -0.13]	-4.74	< .001***
PRMT1	ATG9A	-0.26	[-0.35, -0.17]	-5.41	< .001***
PRMT1	TSC1	-0.25	[-0.34, -0.16]	-5.19	< .001***
PRMT1	ULK1	-0.29	[-0.38, -0.20]	-6.26	< .001***

F

Parameter1	Parameter2	r	95% CI	t(349)	p
PRMT1	HMMR	0.30	[0.21, 0.40]	5.97	< .001***
PRMT1	ESCO2	0.35	[0.26, 0.44]	6.99	< .001***
PRMT1	CHAC2	0.37	[0.27, 0.46]	7.40	< .001***
PRMT1	NUDT6	0.27	[0.17, 0.36]	5.22	< .001***

Figure S7

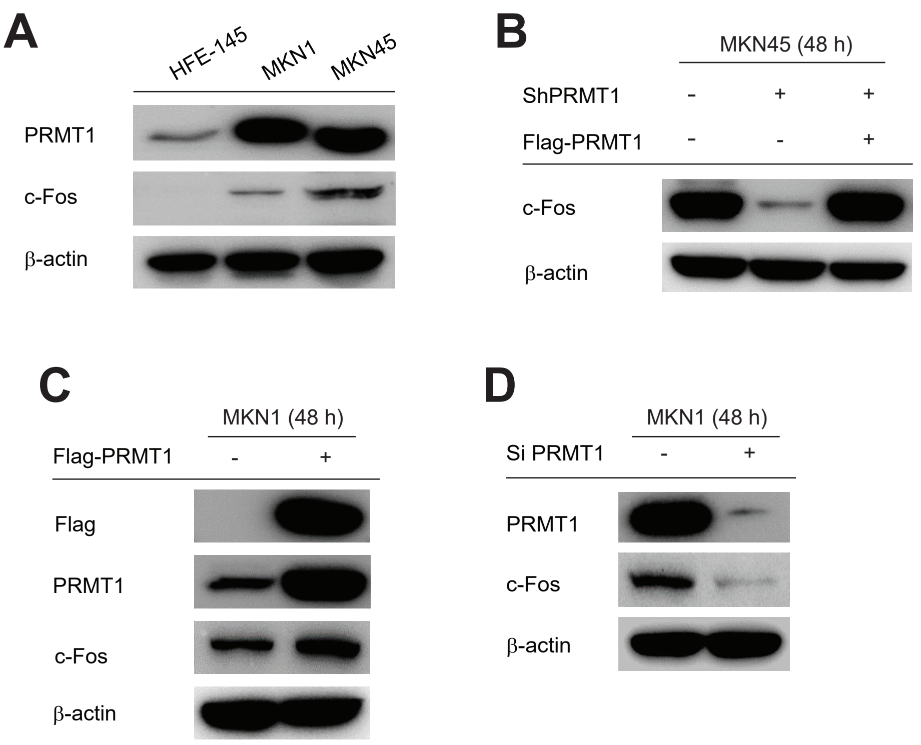

Figure S8

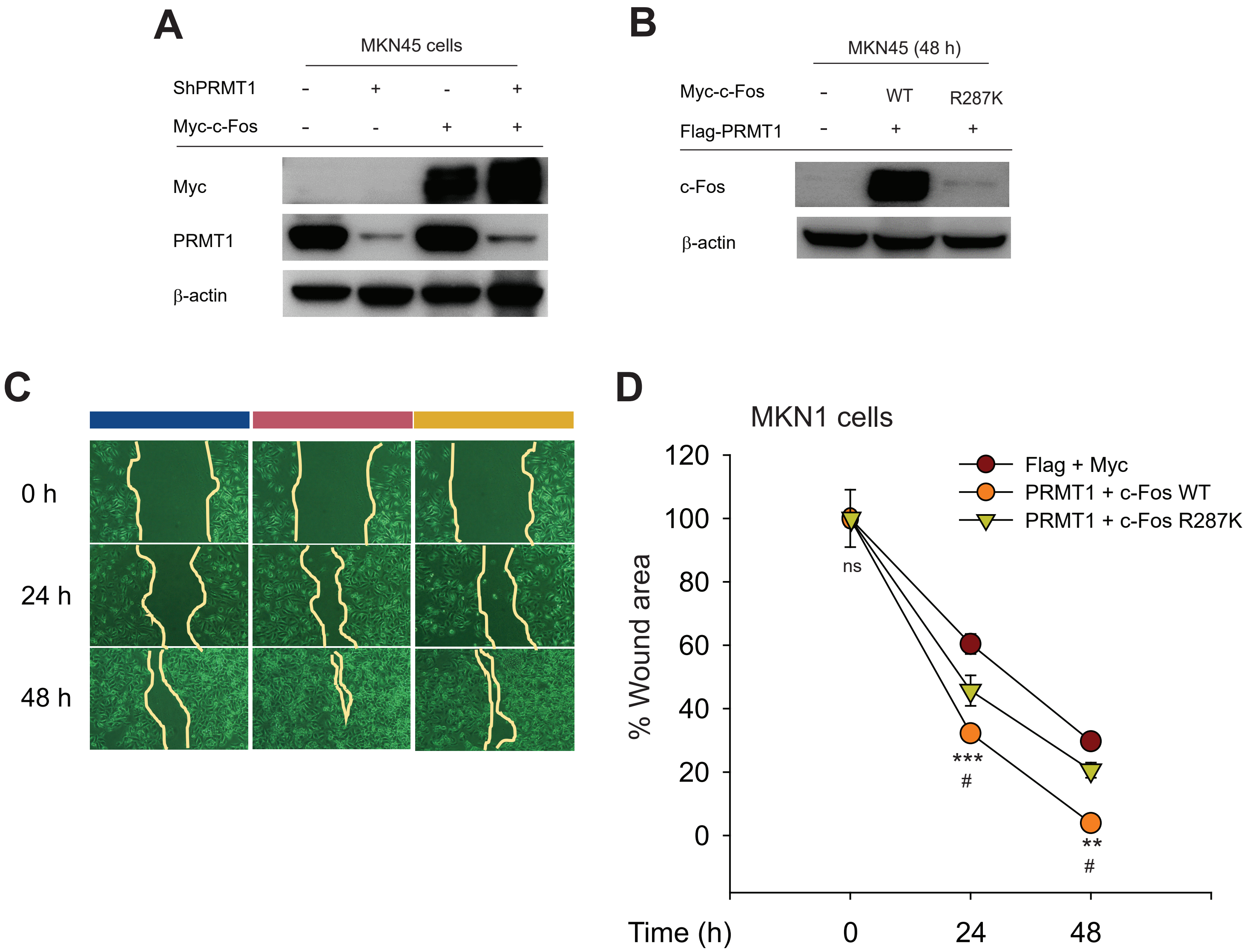

Figure S8

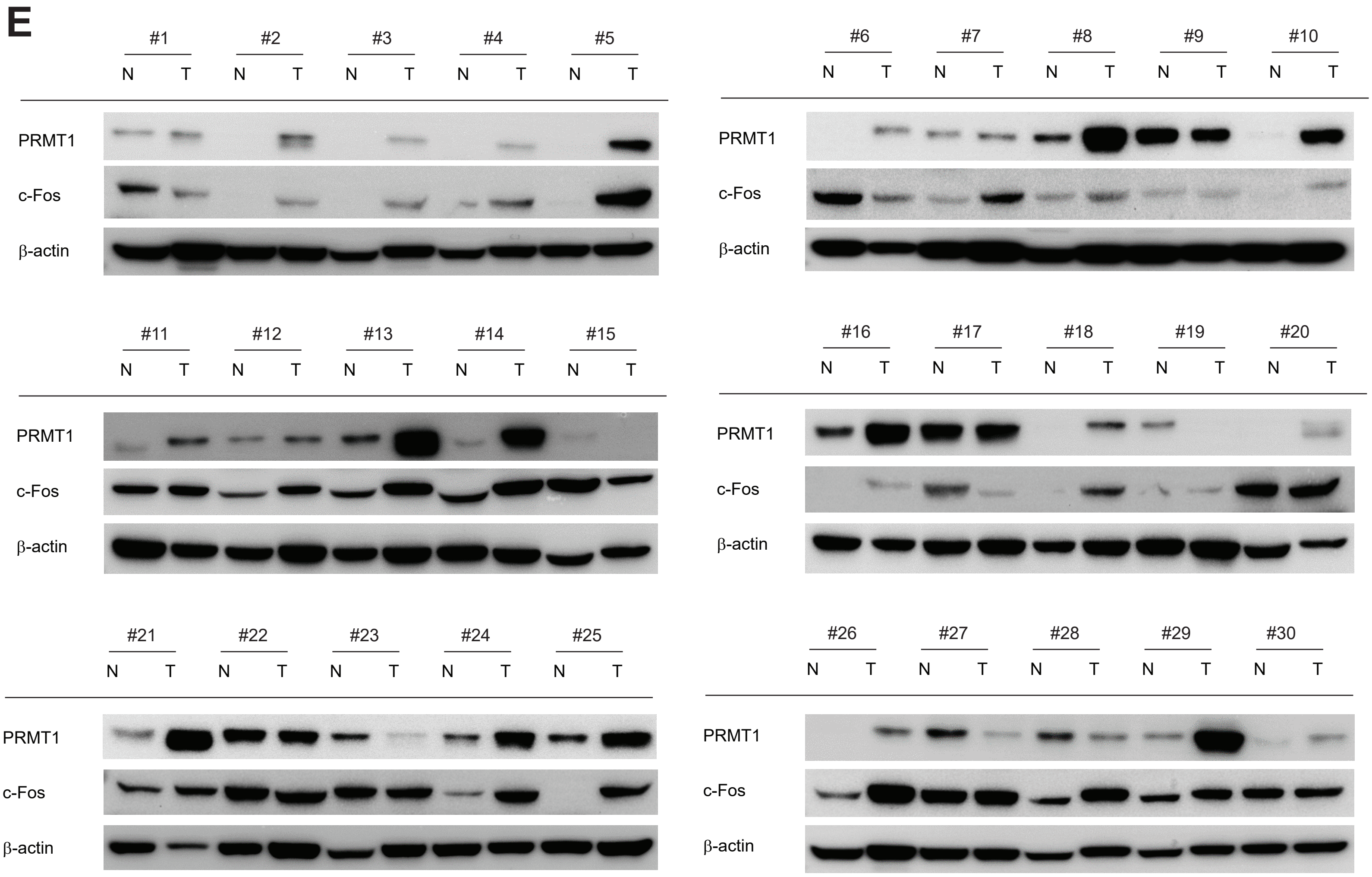
E
#1 #2 #3 #4 #5
N T N T N T N T N T
#6 #7 #8 #9 #10
N T N T N T N T N T
PRMT1
c-Fos
β-actin
#11 #12 #13 #14 #15
N T N T N T N T N T
#16 #17 #18 #19 #20
N T N T N T N T N T
#21 #22 #23 #24 #25
N T N T N T N T N T
#26 #27 #28 #29 #30
N T N T N T N T N T
